# Coherently parallel fiber-optic distributed acoustic sensing using dual Kerr soliton microcombs

**DOI:** 10.1126/sciadv.adf8666

**Published:** 2024-01-19

**Authors:** Jian-Ting Li, Bing Chang, Jun-Ting Du, Teng Tan, Yong Geng, Heng Zhou, Yu-Pei Liang, Hao Zhang, Guo-Feng Yan, Ling-Mei Ma, Zeng-Ling Ran, Zi-Nan Wang, Bai-Cheng Yao, Yun-Jiang Rao

**Affiliations:** ^1^ Fiber Optics Research Center, Key Laboratory of Optical Fiber Sensing and Communications (Education Ministry of China), University of Electronic Science and Technology of China, Chengdu 611731, China.; ^2^ Research Centre for Optical Fiber Sensing, Zhejiang Laboratory, Hangzhou 310000, China.

## Abstract

Fiber-optic distributed acoustic sensing (DAS) has proven to be a revolutionary technology for the detection of seismic and acoustic waves with ultralarge scale and ultrahigh sensitivity, and is widely used in oil/gas industry and intrusion monitoring. Nowadays, the single-frequency laser source in DAS becomes one of the bottlenecks limiting its advance. Here, we report a dual-comb–based coherently parallel DAS concept, enabling linear superposition of sensing signals scaling with the comb-line number to result in unprecedented sensitivity enhancement, straightforward fading suppression, and high-power Brillouin-free transmission that can extend the detection distance considerably. Leveraging 10-line comb pairs, a world-class detection limit of 560 fε/√Hz@1 kHz with 5 m spatial resolution is achieved. Such a combination of dual-comb metrology and DAS technology may open an era of extremely sensitive DAS at the fε/√Hz level, leading to the creation of next-generation distributed geophones and sonars.

## Teaser

Dual-comb enables ultrasensitive and long-distance fiber-optic distributed acoustic sensing with natural fading suppression.

## INTRODUCTION

Distributed optical fiber sensors offer a powerful tool to continuously inform a map of natural variables, providing a unique ability for environmental monitoring, along an optical fiber with a sensing length of tens to hundreds of kilometers (*1*). Among the wide variety of distributed fiber sensing technologies, phase-sensitive optical time-domain reflectometry (Φ-OTDR) based on Rayleigh backscattering stands out as the basis for distributed acoustic sensing (DAS), providing a revolutionary way for highly sensitive measurement of acoustic waves, vibrations, and disturbances (such as intrusions) along a long fiber link with a sensing capacity of up to a million points (*2*). As an irreplaceable sensing technology for fully distributed acoustic/seismic/vibration detection, DAS has demonstrated remarkable advancements covering a wide range of applications, including seafloor faults and ocean dynamics illumination (*3*), earthquake and ocean dynamic detection (*4*, *5*), glacial activity (*6*), gas/oil exploration (*7*), hydrophones (*8*), intrusion detection in energy transportation (*9*), and dynamic structural health monitoring (*10*).

In pursuit of the ultimate performances of DAS, remarkable progress has been made in recent years, such as using functionalized sensing cables (*11*), optical amplification schemes (*12*), pulse coding strategies (*13*), and signal processing algorithms (*14*). Nowadays, the commonly used single-frequency light source becomes one of the bottlenecks (*15*): (i) for a high-sensitivity DAS with relatively short sensing distance, the main limitation lies in the phase noise of the laser source, which cannot be distinguished from the phase changes along the sensing fiber; (ii) for a DAS with long sensing distance, both the sensitivity and the maximum detectable distance are primarily limited by the signal-to-noise ratio (SNR), which is dependent on the laser energy; however, the laser power cannot be infinitely amplified due to the nonlinear effects in fiber; (iii) in all Φ-OTDR–based DAS schemes, the coherent fading caused by the single-frequency laser is also a critical issue.

Soliton microcomb is a promising light source for high-precision metrology, including DAS, thanks to its multi-frequency output with naturally high repetition rate and high coherence at chip scale (*16*–*18*). Over the past two decades, the advancement of microcomb technology has enabled versatile out-of-lab advances in a wide range of applications, such as optical communications (*19*–*21*), photonic machine learning (*22*, *23*), quantum optics (*24*), astrophysical calibration (*25*, *26*), opto-mechanical oscillation (*27*), optical coherence tomography (*28*, *29*), optical frequency synthesizer (*30*), optical atomic clock (*31*, *32*), light detection and ranging (LiDAR) (*33*), and biochemical sensing (*34*, *35*). In addition, the frequency synthesis based on dual combs further offers a tool for converting optical frequencies down to radio frequencies. This technique enables the illustration of broadband spectra while avoiding the need for large optical spectrometers with moving parts and limited optical bandwidth (*36*, *37*). It provides unique high resolution in heterodyne measurements for applications such as ranging, force detection, gas spectroscopy, ultrasonic detection, and photoacoustic imaging (*38*–*42*). Recently, researchers have discovered that by leveraging electrically modulated optical combs, it is possible to enhance scanning speed and spatial resolution for both Brillouin optical time domain analyzer (BOTDA) and Φ-OTDR (*43*, *44*). These advances are impressive and effective attempts. Related to electrically modulated combs, soliton microcombs produced in an all-optical manner can generate more comb teeth over a broader span (reaching up to tens of terahertz) and exhibit higher repetition rates (up to the terahertz level). This capability provides an all-optical tool for distributed fiber sensors, enabling richer frequency multiplexing and larger modulation bandwidth. However, as of now, soliton microcomb–based DAS remains untapped.

Here, for the first time, we demonstrate a coherently parallel DAS based on integrated dual-soliton microcombs. A pair of Kerr soliton frequency combs (repetition rate ≈ 110 GHz) generated in two silicon nitride microrings with a 200-MHz repetition difference forms a dual-comb interferometer. One of the combs serves as the probing light, while the other one works as the local reference, enabling convenient heterodyne measurement without requiring optical spectrum analyzer or high-speed photodetector. In this scheme, multiple-frequency channels with locked phase are simultaneously utilized, allowing them to share one modulator and offer unprecedented SNR. Specifically, the single-sideband phase noise of the probe-local beats is down to −116 dBc/Hz@100 kHz, while relative intensity noise reaches −127 dBc/Hz@100 kHz. This ensures ultrahigh sensitivity for DAS measurement. Moreover, through ingenious design of the dual-comb frequency offset of the 10 comb lines, we avoid the modulation-induced spectrum aliasing, and achieve frequency division multiplexing–based parallel sensing. The response of each frequency channel can be linearly accumulated, demonstrating a 10-fold sensitivity enhancement compared to a single-frequency laser. Experimentally, in a dual-comb–based DAS with a 10-km sensing distance and a 5-m spatial resolution, we obtain world-class sensitivity for acoustic wave detection, which is down to several hundreds of fε/√Hz. Moreover, we find that the dual-comb–based DAS scheme enables higher power delivery in a long fiber with a higher Brillouin threshold; this leads to the expansion of the maximum sensing distance of our DAS from 43 km to 72 km, without requiring any distributed amplification.

## RESULTS

**Fig. 1.**
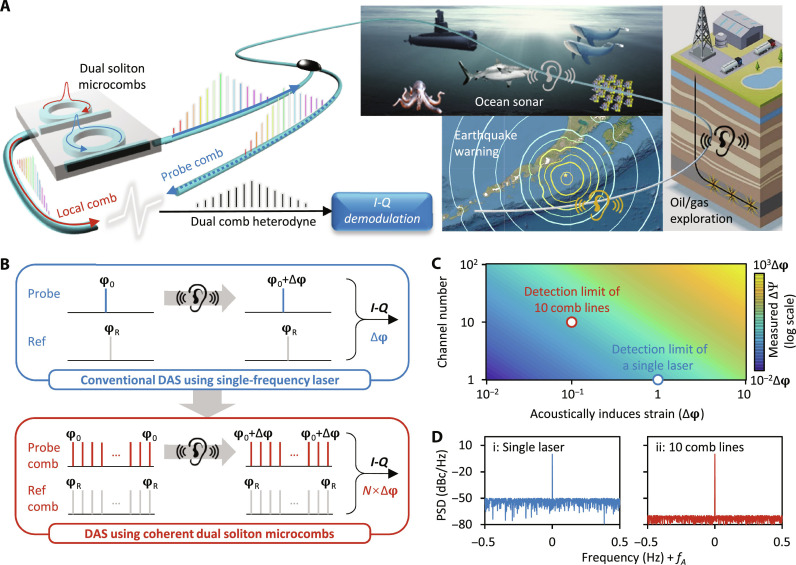
Concept of the dual-soliton microcomb–based DAS. (**A**) Operation of the fiber DAS using dual-soliton comb sources. Two synchronized Kerr solitons with distinct repetition frequencies serve as the probe light and the local reference respectively. The coherently parallel channels are *I*-*Q* demodulated in wavelength division multiplexing heterodyne. (**B**) Principles to obtain higher sensitivity. For conventional DAS using a single-frequency laser, one can only obtain phase alteration due to acoustic signals at one frequency. However, for dual-comb–based DAS, acoustic signal changes optical phase at every comb frequency; thus, one can accumulate them, enabling *N*-fold sensitivity enhancement. (**C**) Correlation of the acoustic signal–induced phase change, the number of comb lines, and the measured phase change. Detection limit of the dual comb scheme is much lower due to the signal accumulation. (**D**) Calculated PSD spectra, when using a single-frequency laser (blue) and 10 comb lines (red).

Figure 1A illustrates the schematic concept of the coherently parallel DAS. Conventionally, a DAS system is driven by utilizing a highly stable single-frequency laser (probe light) and a frequency modulator (local reference). In our dual-comb–based DAS scheme, the probe comb and the local reference comb are independently generated in two microring cavities on chip, using a single external cavity laser diode; both the combs provide multiple frequencies. Because of the soliton nature, the initial phases of all the comb lines are locked, allowing for frequency multiplexing without superimposing noises. The backward Rayleigh scattering of every probe comb line containing the acoustic information beats with the local comb lines one by one at a photodetector, forming a radio frequency (RF) comb via heterodyne. Subsequently, the RF comb is *I*-*Q* demodulated to obtain power density spectrum of the phase change (*45*).

The sensitivity enhancement of the dual-comb–based DAS is explained in Fig. 1B. First, we consider the case of using one single optical frequency. As aforementioned, acoustic signals induced Δφ could be easily measured from the *I*-*Q* demodulation (*46*). In the case of multiple frequencies based on the dual comb, each comb line carries the same acoustic information Δφ. The Δφ detected from each comb line would be finally accumulated after *I*-*Q* processing. In this process, the coherent nature of the dual-soliton microcombs ensures that all the comb lines have the same phase instability. For a number of comb lines *N*, without considering the noise superposition, the dual-comb–based DAS demonstrates *N*-fold sensitivity enhancement. In a DAS system, Δφ linearly depends on the dynamic strain of the optical fiber caused by acoustic signals. Therefore, a dual-comb–based DAS demonstrates the capability to detect weaker acoustic signals. Figure 1C shows the calculated results. Assuming that the detection limit of each frequency channel is Δφ, increasing the number of frequency channels in a dual-soliton microcomb source improves sensitivity due to signal accumulation. In sensing application, the detection limit decreases from Δφ to Δφ/*N*, when the noise base remains constant.

We note that this performance can only be achieved using a fully coherent light source, rather than relying on multiple independent laser sources and modulators. Specifically, when utilizing a single-frequency laser with a noise base of *n*, the SNR for acoustic detection is Δφ/*n*. When using *N* independent optical lasers, apart from the disadvantages in system complexity and device cost, the total phase alteration becomes *N*Δφ while the total noise becomes ∑^*i* = 1:*N*^*n_i_*. After averaging *N* times, the maximum SNR can reach √*N*(Δφ/*n_i_*). However, in the case of using the fully locked dual-soliton microcombs, all the comb lines can be demodulated simultaneously during the *I*-*Q* process with frequency offset correction, resulting in an SNR of *N*Δφ/*n*. Theoretical analysis is provided in note S1. Figure 1D illustrates the power spectral density (PSD) using a single-frequency laser and using 10 comb lines, respectively. In this simulation, we assume *N* = 10 and the detected acoustic phase alteration is Δφexp(2π*f_A_t*) + *n*. Here, *f_A_* is the acoustic frequency, while *n* = 10^−5^Δφ is the random noise.

**Fig. 2.**
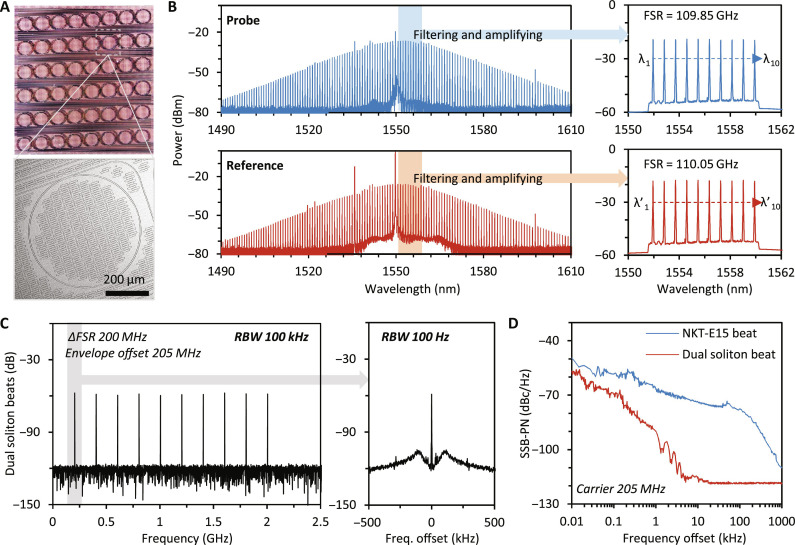
Integrated dual-soliton microcomb source. (**A**) Microscopic pictures of the microresonator for comb generation, a pair of ≈110-GHz combs are formed in two silicon nitride microrings (diameter 460 μm, sectional size 2000 nm by 800 nm). For practical application, the chip is package-encapsulated, with external cavity pump lasers. (**B**) Optical spectra of the dual-soliton (blue: probe, red: reference) microcombs, covering the band 1490 nm to 1550 nm. For DAS measurement, we use 10 comb lines (*u* = 1 to 10). The selected 10 comb lines in each microcomb source are filtered out and amplified to the same power (up to 200 mW each line, limited by the EDFA). We mark the comb frequencies *f*_1_ to *f*_10_ for the probe, while *f*′_1_ to *f*′_10_ for the reference. (**C**) Heterodyne beating of the two filtered combs show the repetition difference Δ*f_rep_* 200 MHz. Zoom-in: each electrical comb line shows SNR > 50 dB with linewidth in the Hz level. (**D**) Measured phase noises of the first dual-soliton pair beat (red) and the self-beat of the NKT-E15 (blue).

Figure 2 characterizes the dual-soliton microcombs. First, we present the microscopic pictures of our on-chip microrings used for soliton comb generation (Fig. 2A). The sectional size of the silicon nitride waveguide is 1650 nm by 800 nm, allowing for single mode oscillation in the anomalous dispersion region around 1550 nm (*D*_2_ = 0.8332 MHz). Here, we use two silicon nitride rings for exciting the probe comb and the reference comb, respectively. The measured *Q* factor of both the two microrings is 5 × 10^6^. The diameters of the two microrings (≈600 μm) are slightly different, enabling dual comb generation with different repetition rates. More detailed information about the microcavities is provided in note S2. By utilizing a tunable laser and a frequency shifter, we generate two dissipative Kerr soliton microcombs, as Fig. 2B shows. In the spectrum, both the two soliton combs demonstrate an envelope in sech^2^ fitting, verifying their soliton state. The repetition frequency of the probe soliton is 109.85 GHz, while the repetition frequency of the reference soliton is 110.05 GHz. To cater to the DAS requirements, we apply a filter to select 10 lines (longitude mode number 2 to 11) from each comb and shape them to an equal power level. In this figure, we also show the zoomed-in spectra. These 20 comb lines are used in the DAS later. By tuning the frequency shifter, we control the spectral difference of the first probe comb line (λ_1_) and the first reference comb line (λ′_1_) at 1.64 p.m., corresponding to 205 MHz.

We plot the beat note of the two combs in Fig. 2C. The repetition difference of the two combs is 200 MHz, and the first beat note appears at 205 MHz. By zooming-in the radio-frequency spectrum, we observe that the SNR of each beat note exceeds 50 dB, and the measured linewidth of each beat note is smaller than 10 Hz, limited by the resolution of our spectrum analyzer. Additionally, we illustrate the single-sideband phase noises (SSB-PN) in Fig. 2D. The blue curve represents the SSB-PN of the self-beat note of the NKT-E15 (with a frequency of 205 MHz), while the red curve represents the SSB-PN of the first beat note of the dual combs, which is also located at 205 MHz. The SSB-PN of the dual comb beats approach −56.7 dBc/Hz @ 10 Hz, −89.8 dBc/Hz @ 1 kHz, and −115.7 dBc/Hz @ 10 kHz, after stabilization. In the acoustic frequency region (ranging from Hz to hundreds of kHz), the dual comb heterodyne offers much lower phase noises (*47*). In practice, the dual-soliton microcomb source could be instrumentally packaged and automatically generated/restarted via the feedback-based laser scanning program. More details are shown in note S2.

**Fig. 3.**
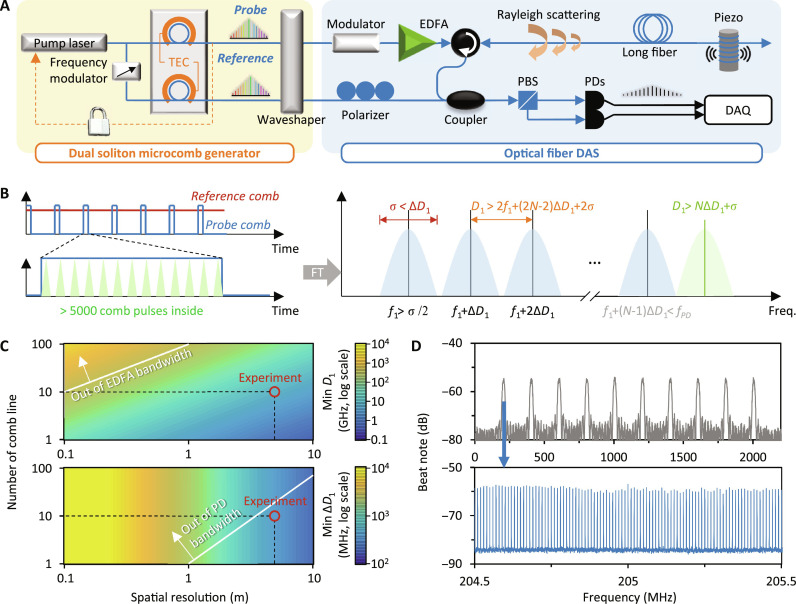
Parametric design of the dual-comb–based DAS. (**A**) Detailed experimental setup of the dual-comb–based DAS. Here, two synthesized soliton microcombs are generated by one single-pump laser in two microcavities with minor different repetition rates, for ensuring the dual-comb interferogram but minimized synchronous noise. The probe comb is filtered and amplified before sensing. Acoustic signals are offered by using a PZT with ≈10-m-long optical fiber winding. Beat notes of the dual comb are hybrid demodulated. (**B**) For realizing distributed sensing, the probe comb is primarily modulated in square waveform (pulse width 50 ns, repetition 0.1 ms in our experiment). In each modulated pulse, there are >5000 comb pulses (*D*_1_ 109.85 GHz). Because of the modulation, sidebands appear around every dual-comb beat note. For avoiding spectral overlapping and meeting the device bandwidths, one needs to carefully design the *D*_1_ and the Δ*D*_1_. (**C**) Calculated parametric spaces. For our DAS system with 5 m spatial resolution (pulse width 50 ns), the comb pair with *D*_1_ ≈ 110 GHz repetition while Δ*D*_1_ = 200 MHz is an optimized choice. Here, the white lines mark the bandwidths of the EDFA and the photodetector. (**D**) Measured dual-soliton beat notes after modulation. They demonstrate separate bands with an interval of 200 MHz. In the zoomed-in panel, we see the modulation period 10 kHz (corresponding to the sensing distance 10 km).

Figure 3 demonstrates the sensing operation. In the experimental setup (Fig. 3A), we utilize a tunable single-frequency laser (NKT E15) to directly generate the probe comb. Simultaneously, we apply a tunable frequency shifter (e.g., an AOM) to the same laser for generating the local reference soliton comb. This configuration allows us to independently tune the repetition rates of the two soliton combs, which are generated in separate cavities. Additionally, since both soliton combs are driven by the same pump laser, this arrangement helps suppress the pumping noise during the heterodyne process. In the DAS system, square pulse modulation plays a crucial role in determining the sensing distance (*L_D_*) and the spatial resolution (*d*). Specifically, *L_D_* is determined by the modulation period (*T_M_*), writing 2*L_D_* = *vT_M_*; here, *v* ≈ 2 × 10^8^ m/s is the light velocity in fiber. Besides, *d* relies on the modulated pulse width τ, given by the equation 2*d* = *v*τ. For instance, we can design *L_D_* = 10 km and *d* = 5 m for common applications. This requires setting the modulation parameters *T_M_* = 0.1 ms and τ = 50 ns. In our system, only one modulator is required to shape the waveforms of multiple comb lines with distinct optical frequencies. After modulation, the probe comb trace is then amplified by using a low-noise erbium-doped fiber amplifier (EDFA) and transmitted through the 10-km-long fiber. This amplification boosts the power of every comb line to the milliwatt level without introducing serious amplitude noise. At a location 5 km away from the probe, we use a standard optical fiber-wound piezoelectric transducer (PZT) to generate an acoustic signal. The signal frequency can be tuned over the range of 1 to 10 kHz. The induced strain in the fiber by the PZT is 11.2 nε/V.

Now, we discuss the parameters of the comb source. Figure 3B illustrates the schematics of the modulated comb lines. In time domain, different from a single-frequency laser, the soliton comb generates ultrafast pulses. Assuming that the repetition rate of the probe comb is *D*_1_, each square wave would consist of τ × *D*_1_ pulses. First, to ensure that the modulated waveform approximates the ideal periodic square wave and to avoid the aliasing effect induced by the sampling of comb pulses, the pulse density within each square envelope should be sufficiently high, with *D*_1_ > *N*Δ*D*_1_ *+* σ; here, *N* is the number of comb lines, and σ represents the spectral bandwidth determined by modulation. In our experiment, each 50-ns square wave contains approximately 5500 comb pulses. More detailed theoretical discussion is provided in note S1. Second, to avoid spectral aliasing induced by modulation, it is necessary to ensure Δ*D*_1_ > σ. Additionally, to prevent aliasing in dual-comb heterodyne, Δ*D*_1_ should be small enough, meeting *D*_1_ > 2*f*_1_ *+* (2*N* − 2)Δ*D*_1_ *+* 2σ, where *f_1_* is the first dual-comb beating frequency. Furthermore, the bandwidth limit of our photodetector (*f*_PD_) needs to be taken into consideration, which means the frequency of the last dual-comb beat note should be smaller than *f*_PD_. This can be expressed as *f*_1_ *+* (*N* − 1) × Δ*D*_1_ + σ/2 < *f*_PD_. Last, in optical band, all the comb lines within the span *N* × *D*_1_ should be located within the effective amplification region of the EDFA.

In Fig. 3C, we show the calculated parametric spaces of the dual-comb–based DAS, indicating the acceptable minimum comb repetition (*D*_1,min_) and the minimum dual-comb repetition difference (Δ*D*_1,min_). We also highlight the inherent limitations of our devices in the experiment, for instance, *f*_PD_ is 2.5 GHz, and the low noise amplification bandwidth of our EDFA is approximately 17 THz (see note S2). The dots on the plot mark the configuration where 10 comb lines are used. Keeping the 5-m spatial resolution, the *D*_1_ of our microcomb is approximately 110 GHz > *D*_1,min_ ≈ 3.6 GHz, while Δ*D*_1_ = 200 MHz > Δ*D*_1,min_ ≈ 188.4 MHz, referring to the square wave modulation. We just use 10 comb lines, mainly considering the optical bandwidth of the EDFA (it limits *N* < 16) and the electrical bandwidth of the low noise photodetector (it limits *N* < 12). As aforementioned, increasing the comb line number would improve the sensitivity in principle. When a further larger comb line number is in-need, one can use a dual-comb source with smaller *D*_1_ and a photodetector with broader bandwidth, or just reduce the spatial resolution. Figure 3D plots the measured beats of the modulated probe comb and the reference comb. It verifies that the sideband of every frequency channel would not overlap with each other. By zooming-in the center of a frequency channel, the modulated structures with 10-kHz interval are obvious. In the future, maintaining the nonaliasing condition while reducing the *D*_1_ and increasing the *f*_PD_, one can use more comb lines. For instance, if *D*_1_ = 10 GHz, Δ*D*_1_ = 50 MHz, and *f*_PD_ > 5 GHz, the number of usable comb lines could be >100. We also note that using locked opto-electrical dual combs is also a viable option. However, considering the above trade-offs of the comb line number, *D*_1_ and Δ*D*_1_, the dual-soliton comb scheme proposed retains its uniqueness and advantages, such as the ability to offer more comb lines and a higher *D*_1_.

**Fig. 4.**
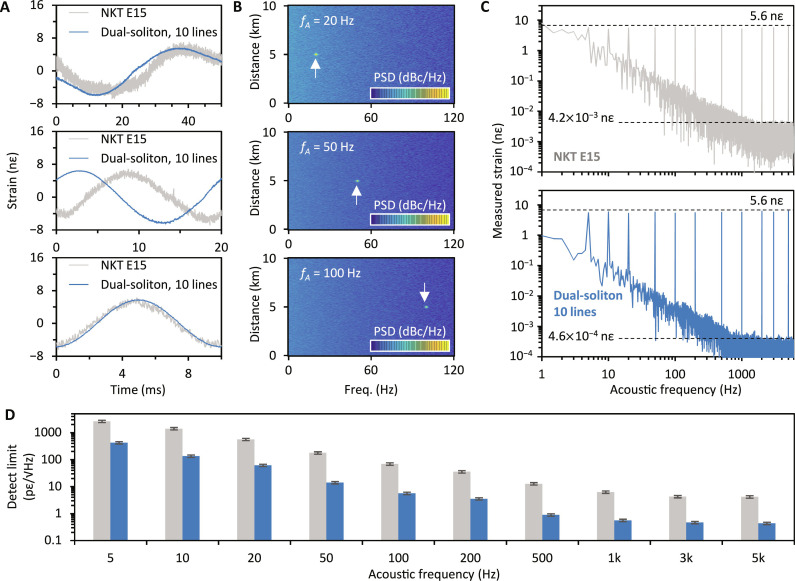
Sensitivity enhancement of the dual-comb–based DAS. (**A**) From top to bottom, temporal DAS traces obtained by using a single-frequency laser (NKT-E15, gray) and the dual-soliton microcomb (blue), at acoustic signal frequencies, 20 Hz, 50 Hz, and 100 Hz. Signal amplitude: ±5.6 nε. (**B**) PSD maps of the three measurements. The bright dot in each map shows the signal. Scale bar, −80 to 0 dBm. (**C**) Sensitivity comparison of the two schemes, with a fixed strain ±5.6 nε at various frequencies. Averagely, for any signal frequency, the strain-to-noise ratio of the dual-soliton scheme is 10 dB higher than the single-frequency laser scheme. Here, we gather the sensing signals from 5 Hz to 5 kHz. (**D**) Detection limit. Gray columns show the results measured by using the single-frequency laser source, while blue columns show the results measured by using the dual-soliton microcombs. For >500-Hz signals, the dual-comb–based DAS approaches the sub-pε/√Hz level. Error bars: repeated measurement uncertainty.

Figure 4 demonstrates the measured results of acoustic sensing. At the location 5 km away from the optical source, we introduce acoustic signals via the PZT with tunable frequencies ranging from 10 Hz to 3 kHz. Figure 4A shows three temporal traces as examples. From top to bottom, the acoustic frequencies are 20, 50, and 100 Hz, respectively. In all the measurements, the strain is fixed at ±5.6 nε. Here, we compare the results using a single-frequency laser (NKT E15, gray curves) and the dual-soliton microcombs (blue curves). The noise in the dual-comb–based measurement is obviously lower, offering higher SNR. Correspondingly, the retrieved space-frequency maps of the dual-comb DAS are shown in Fig. 4B. Typically, for an acoustic frequency of 20, 50, and 100 Hz, the measured SNR of the PSD is larger than 39, 52, and 60 dB, suggesting that the detection limit is 134, 61.5, and 5.6 pε, respectively.

In addition to 20, 50, and 100 Hz, we also test acoustic signals with lower and higher frequencies. Given that the background noise in a DAS decreases with acoustic frequencies, the dual-comb DAS could perform better when sensing rapid fluctuations. Figure 4C presents the measured correlations between frequency and strain; here, we compare the spectra of both a single-frequency laser–based DAS and the dual-soliton microcomb–based DAS. In these measurements, the acoustic frequencies range from 5 Hz to 5 kHz. With a fixed strain amplitude of 5.6 nε, the accumulated strain in our dual-comb DAS is 56 nε (10 lines), accompanied by noncumulative noise. Equivalently, this effectively boosts the detection SNR by one order of magnitude. It suggests that the dual-soliton microcomb–based DAS can detect considerably weaker acoustic signals. Figure 4D shows a summary of detection limits at various acoustic frequencies. On one hand, both the single-frequency laser–based DAS and the dual-soliton microcomb–based DAS have better sensing performances in the higher-frequency region, since the system noise naturally decreases with increasing frequency offset. On the other hand, using the same system and detecting the same acoustic signal at the same location, the dual-soliton microcomb source demonstrates 10-dB sensitivity enhancement for any acoustic frequency. Specifically, when the acoustic frequency is higher than 500 Hz, the dual-comb–based DAS approaches a detection limit <1 pε/√Hz. If shorter sensing distances or larger spatial resolution are designed, the detection limit could be even lower.

**Fig. 5.**
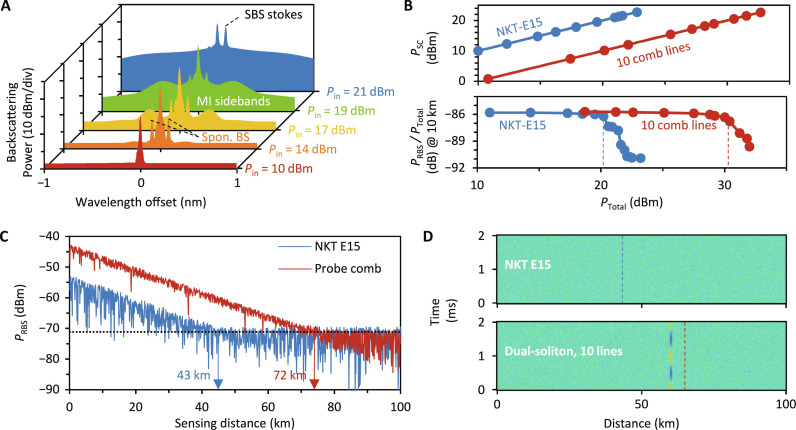
Sensing distance extension by using the frequency comb. (**A**) Backscattering spectral evolution of a single laser channel. The MI and the SBS excitation appears when the input power is high enough. (**B**) Top panel: when increasing the power of a single-frequency channel, the total power of 10 comb lines is 10 times higher. Bottom panel: Rayleigh scattering efficiency decreases fast when SBS is excited. For a single-frequency laser, the deterioration threshold is 20.3 dBm; for 10 comb lines, the deterioration threshold is 30.4 dBm. (**C**) Correlation of the fiber distance and the received Rayleigh backscattering power. For a fixed noise base −70 dB, the dual-comb–based DAS demonstrates a maximum sensing distance of 72 km. (**D**) Comparison of the experimental sensing results. By setting an acoustic signal (1 kHz, ±5.6 nε) at the location 60 km away from the optical source, we show that the single-frequency laser–based DAS cannot detect such a signal, but dual-comb–based DAS can catch this signal accurately.

Moreover, we also find that the comb source does help to improve the sensing distance, since multi-frequency transmission in fiber can provide higher optical power. It is well known that the power of the light source determines the sensing distance, due to the inherent loss in fiber. However, the power of a single-frequency laser cannot be unlimitedly amplified, due to the nonlinear effects such as modulation instability (MI) and stimulated Brillouin scattering (SBS) (*12*). Thanks to the multi-wavelength nature, the total power of a comb source could be much higher due to higher MI and SBS thresholds. Figure 5A shows the measured backscattering spectral evolution of a single-frequency channel in a 10-km single-mode fiber. When increasing the optical power, each frequency comb line (similar to a single-frequency laser) would excite nonlinearities. When the input power (*P*_in_) increases from 11 to 21 dBm, spontaneous Brillouin scattering, MI, and SBS occur. The SBS would seriously consume the input power *P*_in_, and thus suppress the Rayleigh scattering efficiency. Figure 5B illustrates the difference of using a single-frequency laser and 10 comb lines. Because of the frequency multiplexing nature, for every frequency channel with equal power *P*_SC_, the total power of our 10 line comb source can provide a total power *P*_Total_ = 10 × *P*_SC_. Therefore, the nonlinearity threshold of the comb source is 10-fold higher than that of the single-frequency laser. Specifically, the ratio of the Rayleigh backscattering power to the input total power (*P*_RBS_/*P*_Total_) starts to deteriorate when *P*_Total_ > 20.3 dBm for a single-frequency laser–based DAS. However, the Rayleigh efficiency is stable until *P*_Total_ > 30.4 dBm for the comb-based DAS. This indicates that the dual-comb strategy can improve the in-fiber power tolerance to achieve higher SNR.

Figure 5C plots the received Rayleigh scattering power of the DAS system. In our experiment, the total loss in fiber *A* ≈ 0.4 dB/km (round trip), the Rayleigh scattering coefficient *R* ≈ −73 dB, and the noise limited detectable power of our photodetector *P*_PD_ ≈ −70 dBm. The maximum sensing distance is located at the position where the optical signal decays to the detection limit of the photodetector, given by *L*_max_ *=* (*P*_Total_ *− R − P*_PD_)*/A*. More analysis and calculations are shown in note S1. For a single-frequency laser–based DAS, by carefully controlling the launched-in optical power up to 20.3 dBm (below the SBS threshold), we obtain a maximum sensing distance of 43 km. When using the probe comb (10 lines), the SBS-free *P*_Total_ can reach 30.4 dBm, and the maximum sensing distance reaches 72 km. Although the 72-km sensing distance is shorter than those DAS systems based on distributed amplification (*2*), it is already an improvement for conventional DASs based on single-frequency lasers. It is anticipated that the sensing distance could be further improved by combination with distributed amplification. Finally, we verify the results in the sensing experiment (Fig. 5D), via setting the DAS spatial resolution to 10 m (modulated square wave pulse width, 100 ns) and the fiber length to 100 km (modulated square wave repetition, 1 kHz). We add an acoustic signal (1 kHz, ±5.6 nε) at the location 60 km away from the optical source. The single-frequency laser–based DAS cannot detect such a weak signal, but the dual-comb–based DAS can catch this signal accurately. In note S3, we compare the performances of our work and other advanced DAS schemes.

## DISCUSSION

Here, we report an approach for coherently parallel fiber-optic DAS, leveraging integrated dual-soliton microcombs. The dual-comb light source provides colocked multiple-frequency channels, enabling the linear superposition of sensing signals while concurrently mitigating optical noise sources. Through meticulous control of the repetition frequency difference between the soliton pair, we achieve high-precision phase demodulation using frequency division multiplexing. This scheme demonstrates substantial potential for sensitivity enhancement. In the experiment, by utilizing a configuration with 10 comb lines, we obtain a detection limit down to sub-pε/√Hz for acoustic signals with frequencies ≥100 Hz. Additionally, this frequency multiplexing capability contributes to effective coherent fading suppression. Furthermore, the comb source’s ability to facilitate SBS-free transmission at watt-level power in optical fibers extends the sensing distance. Such a combination of dual-soliton microcombs and DAS not only illustrates a physical paradigm expanding the utility of microcombs into the realm of optical fiber sensing, but also introduces a groundbreaking strategy to surpass the performance limitations encountered by current fiber-optic DAS systems, which were typically constrained by single-frequency laser sources. In the future, by increasing the number of comb lines and optimizing the spectral parameters, the potential of the dual combs and optical fiber sensing synergy can be further enhanced for wider applications.

## MATERIALS AND METHODS

### Design and fabrication of the comb device

In a silicon foundry, we fabricated high-*Q* silicon nitride microresonators with measured loaded *Q* over 5 million and FSR ≈ 110 GHz in a 460-μm-diameter ring structure. The nitride ring has a 2000-nm by 800-nm cross section and a 200-nm gap to the input-output coupling waveguide. The microring is encapsulated in a top oxide cladding. On a 5-mm by 5-mm chip, we integrated 64 ring devices. By precisely controlling the temperatures of the rings (temperature difference, 37°C) in two chips, we realized that FSRs of two rings vary slightly. Here, we chose the FSR difference ≈ 200 MHz for dual microcomb generation. To ensure the soliton stability, an integrated auxiliary laser (Pure Photonics PPCL 550) was first tuned into the blue-detuning region of a cavity mode, and subsequently a pump laser (NKT E15) was red-tuned in another cavity mode. By properly setting the power and detuning the pump laser (200 mW) and auxiliary laser (200 mW), the heat flow caused by the lasers can be balanced, allowing deterministic single soliton generation.

### Fiber-optic DAS and data processing

In the DAS measurements based on coherently parallel multiple-frequency channels, we modulated the probe comb for spatial information acquisition, while using the reference comb for heterodyne demodulation with a single photodetector. Related to the ultrahigh repetition of soliton pulses (110 GHz), the modulation with 1 to 10 kHz is much slower. Thus, each frequency channel can be regarded as continuous wave. Square wave modulation enables spatial identification along the optical fiber. For fulfilling the 10- to 100-km sensing, an EDFA is essential for probe light amplification, but below the nonlinear threshold. The fiber of the DAS is a typical single-mode fiber. We used a fiber-wound PZT to generate acoustic oscillation, with tunable oscillation frequency ranging from 1 to 10 kHz, with strain in fiber 11.2 nε/V. We measured the backward Rayleigh scattering temporally. The phase alteration difference of the probe and the reference combs were analyzed in the frequency down conversion based on the *I*-*Q* demodulation scheme. By using the Fourier transform conveniently, we studied the power spectral density of the acoustic responses with high resolution.

## Supplementary Materials

### This PDF file includes:

Supplementary Text Figs. S1 to S10 Tables S1 to S4 References
