# Supplementary material for: Coherently parallel fiber-optic distributed acoustic sensing using dual Kerr soliton microcombs
Source: Sci Adv. 2024 Jan 19;10(3):eadf8666. doi: 10.1126/sciadv.adf8666 (PMC10798552; doi:10.1126/sciadv.adf8666)
Supplement: Supplementary file 1 — Supplementary Text Figs. S1 to S10 Tables S1 to S4 References [file sciadv.adf8666_sm.pdf]

Science Advances
AAAS

Supplementary Materials for

Coherently parallel fiber-optic distributed acoustic sensing using dual Kerr soliton microcombs

Jian-Ting Li *et al.*

Corresponding author: Yun-Jiang Rao, yjrao@uestc.edu.cn; Bai-Cheng Yao, yaobaicheng@uestc.edu.cn

Sci. Adv. **10**, eadf8666 (2024)
DOI: 10.1126/sciadv.adf8666

This PDF file includes:

Supplementary Text
Figs. S1 to S10
Tables S1 to S4
References

Supplementary Text

Supplementary Note S1. Extended theoretical discussion

Model of soliton Kerr comb generation has been widely discussed (*48*). In the soliton comb formation, a considerable issue is the thermal balance, which limits the stability, robustness of a soliton comb device in application. In this work, we utilize the auxiliary laser heating scheme to obtain the deterministic single soliton generation in two microcavities, this strategy makes our dual-comb source very reliable and applicable as an out-of-lab tool.

Considering the auxiliary laser heating scheme, the thermal effect of the microcavity is regulated by injecting the auxiliary laser into the microring. Therefore, the influence of auxiliary light should be considered on the basis of standard LLE. Since the auxiliary light propagates in the opposite direction to the pump light, only a linear phase shift is applied to the forward pump light and Kerr comb through the XPM effect, and the magnitude of the linear phase shift is related to the intracavity auxiliary light power. In addition, in order to simulate the thermal effect regulation of auxiliary light on the microcavity, it is necessary to introduce the thermal nonlinear term. Therefore, the LLE model with a cavity thermal effect and auxiliary laser can be expressed as follows (*49*):

$$T_R \frac{\partial E_c(t,\tau)}{\partial t} = \left[-\alpha - i\left(\delta_p - \delta_{th}\right) + iL\sum_{k\geq 2}\frac{\beta_k}{k!}\left(i\frac{\partial}{\partial \tau}\right)^k + i\gamma L\left(\left|E_c\right|^2 + 2P_a\right)\right] E_c(t,\tau) + \sqrt{\theta P_{pump}} \tag{S1}$$

$$T_R \frac{\partial E_a(t,\tau)}{\partial t} = \left[-\alpha - i\left(\delta_a - \delta_{th}\right) + iL\sum_{k\geq 2}\frac{\beta_k}{k!}\left(i\frac{\partial}{\partial \tau}\right)^k + i\gamma L\left(\left|E_a\right|^2 + 2P_c\right)\right] E_a(t,\tau) + \sqrt{\theta P_{aux}} \tag{S2}$$

$$\frac{\partial \delta_{th}}{\partial t} = \frac{1}{C_p}\left[\alpha_T \frac{Q}{Q_{abs}}\left(P_c + P_a\right) - K\delta_{th}\right] \tag{S3}$$

In above equations, T_R is the roundtrip time of the Si_3N_4 microring; t and τ are denote the slow time at the scale of the cavity photon lifetime and the fast time defined in a reference frame moving at the light group velocity in the cavity, respectively, which are used to describe the evolution of the intracavity comb field $E_c(t, \tau)$ and auxiliary laser field $E_a(t, \tau)$; α is the cavity decay per roundtrip; δ_p and δ_a are the detuning of the pump and auxiliary laser, while δ_{th} is the thermal drifting of the resonance; L is cavity length; γ is the nonlinear coefficient; θ is the coupling rate from the bus waveguide to the microring; β_k is the k-th order dispersion; P_a and P_c are the intracavity average power of pump-comb field and auxiliary laser field, respectively; P_{pump} and P_{aux} are the pump laser power and auxiliary laser power, respectively; α_T is the temperature coefficient, C_p is the thermal capacity, K is the thermal conductivity, Q and Q_{abs} are the loaded and intrinsic quality factors.

In the experiment, the pump laser and the auxiliary laser have the same polarization state, located in the same mode family of the microcavity. So, in the model, α, θ, and β_k are assumed to be identical for the pump and auxiliary lasers. Based on the equations mentioned above, we can analytically calculate the soliton energy, spectrum and waveform evolution versus pump detuning, using the experimental parameters (listed in **Table S1**). **Fig. S1A** demonstrates the comb power evolution over the pump detuning process. The purple and red curves represent the results without auxiliary laser assistance and with auxiliary laser assistance, respectively. It is clear that the soliton steps are very short and it is difficult to obtain the single soliton state stably without the auxiliary laser. However, with the assistance of the auxiliary laser, the simulation results show the long soliton steps, and the single soliton output can be obtained deterministically. **Fig. S1B** maps the evolution of intracavity waveforms (left) and comb spectra (right) under auxiliary-assistance. It shows a reliable approach to deterministically generate single soliton in each microresonator.

Table S1. Parameters used in analytical calculations for soliton microcomb

Parameters	Value
n_{eff}	2.4
L	1.2 mm
D_1	110 GHz
γ	1.0 $w^{-1}\cdot m^{-1}$
β_2	-20×10^{-27} s^2 m^{-1}
α	0.004
θ	0.002
$(\alpha_T Q)/(Q_{abs}C_p)$	5×10^{-6} J^{-1}
K/C_p	7×10^{-4} s^{-1}

Then we analyze the DAS. Typically, for a single frequency channel, temporal trace of the detected backward scattering of probe S_p in DAS is:

$$S_p(t) = E_p \cos\left[\omega_p t + \varphi_p(t) + \Delta\varphi(t)\right] + S_{n,p}(t) \tag{S4}$$

Here E_p is the backward scattering amplitude, ω_p is the probe frequency, $\varphi_p(t)$ is the initial phase of the probe with phase noise, $\Delta\varphi$ is the acoustic signal induced phase alteration, $S_{n,p}(t)$ is the intensity noise of the probe laser. While the local reference trace is written in:

$$S_r(t) = E_r \cos\left[\omega_r t + \varphi_r(t)\right] + S_{n,r}(t) \tag{S5}$$

Here E_r, ω_r, $\varphi_r(t)$, $S_{n,r}(t)$ is the amplitude, frequency, initial phase and intensity noise of the local reference. After low-pass filtering in a band-limited photodetector, heterodyne beat note of the two beams is:

$$S_B(t) = \frac{E_p + E_r}{2} \cos\left[(\omega_p - \omega_r)t + (\varphi_p - \varphi_r) + \Delta\varphi(t)\right] + S_{n,p}(t)S_{n,r}(t) \tag{S6}$$

Here $(\varphi_p-\varphi_r)$ is the reduced phase noise, or flicker noise satisfying the power law (*50*). By using the Hilbert transform, we use a $cos[(\omega_p - \omega_r)t+(\varphi_p-\varphi_r)]$ and a $sin[(\omega_p - \omega_r)t+(\varphi_p-\varphi_r)]$ to further mix the S_B, thus remove the frequency terms, obtaining

$$\begin{aligned} I(t) &= \frac{E_p + E_r}{4} \cos\left[\Delta\varphi(t)\right] + n \\ Q(t) &= -\frac{E_p + E_r}{4} \sin\left[\Delta\varphi(t)\right] + n \end{aligned} \tag{S7}$$

Here n represents the intensity noise related to the $S_{n,p}S_{n,r}$ (*51*),(*52*).Finally we can retrieve the $\Delta\varphi(t) = -arctan[-Q(t)/I(t)]-[4n/(E_p+E_r)]$. Now we consider the case in a dual-comb DAS, where the frequency channels of the probe comb beat with the reference comb one-by-one. Hence one can express the down-mixed RF signal as follows:

$$S_{B-comb}(t)=\sum_{i=1}^{N}\frac{E_{p,i}+E_{r,i}}{2}\cos\left[(\omega_{p,i}-\omega_{r,i})t+(\varphi_{p,i}-\varphi_{r,i})+\Delta\varphi_i(t)\right]+\sum_i S_{n,p,i}(t)S_{n,r,i}(t) \quad (S8)$$

Here N is the number of comb lines. When using multiple single lasers with different optical frequencies simultaneously, every $\varphi_{p,i}$-$\varphi_{r,i}$ is different from each other if they are incoherent, so that their accumulation could be $\sqrt{N}\times(\varphi_{p,i}-\varphi_{r,i})$ in minimum (via white noise averaging), when we use $\cos[(\omega_p - \omega_r)t]$ and a $\sin[(\omega_p - \omega_r)t]$ to mix it in the *i-q* process. But in the case of fully-stabilized dual comb, soliton locking enables $\varphi_{p,1}$-$\varphi_{r,1}$ = $\varphi_{p,2}$-$\varphi_{r,2}$ = ··· = $\varphi_{p,N}$-$\varphi_{r,N}$. = φ_Δ. Besides, when we ignore the group velocity dispersion (actually the comb bandwidth is typically not wide enough), all the $\Delta\varphi_i$ equal to each other, $\Delta\varphi_i = \Delta\varphi$. Moreover, we assume the intensity of each comb line is the same. Therefore, we simplify the above equation to:

$$S_{B-comb}(t)=\sum_{i=1}^{N}A\cos\left[(\omega_{p,i}-\omega_{r,i})t+\varphi_\Delta+\Delta\varphi(t)\right]+\sum_i S_{n,p,i}(t)S_{n,r,i}(t) \quad (S9)$$

Here A is the beating intensity of each probe comb line and its corresponding reference comb line. In this case, we can use *i-q* vectors to process the $S_{B\text{-}comb}$.

$$\begin{aligned}i&=\left[\cos((\omega_{p,1}-\omega_{r,1})t+\varphi_\Delta)\quad \cos((\omega_{p,2}-\omega_{r,2})t+\varphi_\Delta)\quad \cdots\quad \cos((\omega_{p,N}-\omega_{r,N})t+\varphi_\Delta)\right]\\ q&=\left[\sin((\omega_{p,1}-\omega_{r,1})t+\varphi_\Delta)\quad \sin((\omega_{p,2}-\omega_{r,2})t+\varphi_\Delta)\quad \cdots\quad \sin((\omega_{p,N}-\omega_{r,N})t+\varphi_\Delta)\right]\end{aligned} \quad (S10)$$

After ignoring the cross-terms with time-frequency information (by filtering the high frequency components and the DC component), we construct the I-Q matrix,

$$\begin{bmatrix}I_{comb}\\ Q_{comb}\end{bmatrix}=\begin{bmatrix}\frac{A}{2}\cos[\Delta\varphi(t)] & \frac{A}{2}\cos[\Delta\varphi(t)] & \cdots & \frac{A}{2}\cos[\Delta\varphi(t)]\\ \frac{A}{2}\sin[\Delta\varphi(t)] & \frac{A}{2}\sin[\Delta\varphi(t)] & \cdots & \frac{A}{2}\sin[\Delta\varphi(t)]\end{bmatrix}+n \quad (S11)$$

The phase vector $\Psi = \arctan\{-Q_{comb}/I_{comb}\}$. Inner sum of Ψ_i is the final phase alteration, i.e. $\sum\Psi_i = N\Delta\varphi + 2n/A$. Now we understand that the sensitivity of a dual-comb based DAS using N comb lines has N folds sensitivity enhancement compared to a single frequency laser based DAS. Meanwhile, since the total energy of a comb is N folds higher than a single frequency laser, the intensity SNR is also linearly enhanced, as **Fig. S1C** shows schematically.

Specifically, we discuss the noise of the dual-comb based DAS here. First is the light source noise, mainly including the phase noise and the intensity noise. They are induced by the pumping laser, which is inevitable. We commonly characterized the light source noise by using the parameter relative intensity noise (RIN). Each of the microcomb line has the same RIN equal to the pumping laser. But related to using single frequency, the utilization of soliton comb can reduce the effective RIN due to higher energy. In comparison, if using multiple single frequency lasers rather than the locked comb, the total RIN would accumulate to a higher value. Secondly, there is photoelectric detector noise. Photoelectric detector converts optical signals into electrical signals for further processing. While in this detecting process, there is some noise added to the original signal. Unlike the light source noise, the photoelectric detector noise has a relatively fixed power, hence its amplitude keeps when the signal intensity changes. Therefore, we can suppress the photo-detection noise when using comb source because of the increased effective power. Thirdly, the digital signal processing noise. The digital signal processing can introduce random errors. For the φ-OTDR demodulation, we map the inverse trigonometric functions via an unwrap process to obtain the phases. There are two schemes to reduce such noise: One is to increase the sampling rate of signal, and the other one is to improve the SNR of original signal by using the comb source. In **Fig. S1D**, we show the simulated power spectral density of a single frequency laser based DAS and a ten comb-line soliton based DAS. In this simulation, we assume a sinusoidal acoustic signal with frequency 1 kHz. Its maximum $\Delta\varphi$ is normalized, with a fixed noise intensity base -20 dB. For a single frequency laser based DAS, the detected power spectrum shows a 40 dB SNR. For a 10

comb-line-based DAS, the total signal intensity increases 10 dB, but the noise keeps the same. In the same normalized scale, the SNR increases by 20 dB.

Now we discuss the parameters of the comb source used in the DAS system. Typically, to achieve spatial positioning, the laser source must be properly modulated to be square wave pulses, before sending into the long fiber. The square wave shape modulation mainly aims to avoid self-phase-modulation along a long fiber. The modulation parameters determine the maximum sensing distance and spatial resolution. For instance, in a DAS detecting acoustic waves with 0 ~ 10 km distance and 5 m spatial resolution, the modulation period is 0.1 ms, while width of each single square wave pulse is 50 ns. In a conventional single frequency laser based DAS, the energy in every square wave pulse remains constant. But when we use a frequency comb with multiple frequencies, the modulated square wave contains multiple small pulses inside. This situation is similar to 'temporal sampling'. For ensuring the sensing performance, high comb pulse density in every square envelope is important to avoid spectral aliasing. **Fig. S2A** shows the ideal square wave with 50 ns width. By using Fourier transform, the spectrum is in standard sinc shape (**Fig. S2B**). **Fig. S2C** and **Fig. S2D** plot the cases when we use pulsed lasers instead of the single frequency laser (CW). First, when there is only one comb pulse in every square wave (pulse repetition rate equals to the modulation repetition rate, i.e. 10 kHz), its spectrum is a constant, while the modulation information is completely lost. Then, with increasing the pulse density, the temporal trace increasingly approaches the ideal square wave. When the comb pulse density reaches 5001 in one square wave, its spectrum has almost become a standard sinc function, in the ± 50 GHz band. In dual comb heterodyne, every down-mixing comb line after modulation has a spectral bandwidth, in approximation the non-aliasing $\sigma = 3\pi/\tau$, here τ is the width of each square pulse, as **Fig. S2E** shows. Here σ also determines the minimum dual comb repetition rate offset ($\Delta D_{1,min} > \sigma$). Assuming one uses N comb lines, the comb repetition rate must be higher than $N\times\Delta D_1 + \sigma \geq (N+1)\sigma$ (**Fig. S2F**).

Besides sensitivity enhancement, using combs to divide optical energy across different frequencies can help improve the sensing distance. The optical field propagating along a fiber can be simply written as

$$E_o(L) = \sqrt{P_{in}}\exp(i\omega_o \frac{n_{eff}L}{c}) \quad \text{(S12)}$$

Here L is the transmitting distance, P_{in} is the input power at $L = 0$, ω_o is the optical angular frequency, c is the light velocity in vacuum, n_{eff} is the effective refractive index in fiber. In the electromagnetic model, n_{eff} is a complex parameter given by $n_{eff} = n_r + in_i$, here n_r is the real index determining the transmitting phase, while n_i is the imaginary part determining the loss. In a DAS, we measure the backward Rayleigh scattering of the optical field $E_o(L)$. Therefore we write the intensity of the Rayleigh scattering as

$$E_R(L) = r\sqrt{P_{in}}\exp(-\alpha L)\exp(i\beta L) \quad \text{(S13)}$$

Here r is the Rayleigh scattering coefficient, $\alpha = n_i\omega_o/c$ is the loss coefficient, $\beta = n_r\omega_o/c$ is the propagation constant (*2*). When considering the backward scattering power,

$$P_R(L) = \left|E_R(L)\right|^2 = r^2 P_{in}\exp(-2\alpha L) \quad \text{(S14)}$$

In log scale, **Fig. S3A** illustrates the forward transmitting optical power with different inherent losses. **Fig. S3B** depicts the backward scattering Rayleigh power while fixing the fiber loss A = 0.4 dB/km. In our experiment, the maximum input power of the single frequency laser is $P_{in} \approx 20.4$ dBm, while the maximum power of 10 comb lines reaches $P_{in} \approx 30.2$ dBm. In our 10 km DAS, the spatial resolution is 5 m (square pulse width 50 ns), while in our 100 km DAS, the spatial resolution is 10 m (square pulse width 100 ns). Typically, the Rayleigh scattering coefficient is $R \approx$ -73 dB. Based on the above parameters, we calculate the maximum sensing distance in **Fig. S3C**. The blue solid line represents the Rayleigh scattering power using one single frequency laser (input peak power 20.4 dBm), while the red solid line represents the Rayleigh scattering power using 10 comb

lines (input peak power 30.2 dBm). When additional nonlinear losses are considered (e.g. stimulated Brillouin scattering or self-phase-modulation), the optical power in the fiber would decay faster. For instance, the red and dashed lines show the case that the in-fiber loss reaches A = 1.5 dB/km. Finally, the maximum sensing distance is determined by how weak back-scattering power we can detect. This is determined by the sensitivity of the photodetector. Note that the minimum detectable power is P_{PD}, the maximum sensing distance L_{max} can be expressed by the following equation. In **Fig. S3C**, several examples of P_{PD} are shown as colored horizontal lines. **Fig. S3D** illustrates the correlation of A and L_{max}, using the parameters in our experiment: $P_{PD} \approx$ -70 dBm and R = -73 dB.

$$L_{\max} = \frac{P_{in} - R - P_{PD}}{A} \tag{S15}$$

Supplementary Note S2. Extended measurements

Two silicon-nitride (Si_3N_4) microring cavities with a width-height cross-section of 2000×800 nm^2 are utilized for dual frequency combs generation. **Fig. S4A** shows the transmission spectra of the Si_3N_4 microring cavity. By scanning a single-mode laser (short-term linewidth about 300 kHz) with low power through the resonance, we measure the full-width at half-maximum (FWHM) of the Lorentzian-shaped resonance. Low input power levels (typically less than 5mW) are used to avoid thermally induced distortion of the line shape caused by resonant-field build-up within the cavity. The free spectral range (FSR, defined as the wavelength spacing between modes with successive angular mode number) is about 0.883 nm, which corresponds to approximately 110.051 GHz mode-spacing. The inset shows the simulation results of the transverse electrical mode of the microcavity. The sectional geometry ensures the single mode transmission nature. **Fig. S4B** shows details of a single resonance which corresponds to a loaded quality factor of Q ≈ 5×10^6. **Fig. S4C & S4D** demonstrate the transmission spectrum of the other one microring cavity (109.851 GHz, Q ≈ 5×10^6). By carefully tuning the cavity-temperature via a thermal electrical cooler, we can use one single pump frequency to generate two single soliton combs in the two microcavities.

Before comb generation, we measure the performance of the single frequency laser (NKT Koheras BASIK E15), which is used as the pump for soliton excitation. In the sensing, we also compare the performance of the dual-comb based DAS and the single frequency laser based DAS. **Fig. S4E** demonstrates the experimental setup for heterodyne measurement of the single frequency laser. First, we use a 1:9 coupler to divide the line into two propagating paths. The 90% part of the signal passes through a fiber optic delay line (~50km), and the other is modulated by the acoustic-optical modulator (AOM, AOM-GOOCH &HOUSEGO T-M080-0.4C2J-3-F2P) with a frequency shift of 80MHz. Finally, the two signals are coupled back and detected in a photodetector, thus we obtain the heterodyne frequency spectrum after auto-correlation retrieval. **Fig. S4F** plots the laser linewidth of our NKT E-15. The measured linewidth of the NKT E-15 at λ ≈ 1550 nm is 110 Hz. The relative intensity noise (RIN) is obtained by using an electrical spectrum analyzer and an oscilloscope (1.25 GHz bandwidth, 4 GHz sampling rate). In the electrical spectrum analyzer (with an appropriate resolution bandwidth, RBW 10 Hz), we measure the noise of photodetector ($P_0(f)$) under the condition of empty-load. Then, we measure the output signal of NKT E15 in the photodetector, and the voltage average value of the signal ($E[x(t)]$) is obtained by an oscilloscope with the fixed loading resistance R =50 ohms. Finally, we obtain the power spectral density of the signal ($P_{int}(f)$) from the spectrum analyzer with the same RBW. The RIN of the signal is calculated as follows. The measured RIN of NKT E15 is about -87 dB/Hz at 1 Hz offset while about -150 dB/Hz at 100 kHz offset as shown in **Fig. S4G**.

$$RIN = \frac{N}{A} = \frac{\left[P_{\text{int}}(f) - P_0(f)\right]/RBW}{E\left[x(t)\right]^2/R} \tag{S16}$$

Figure S5A shows the experimental architecture of dual Kerr soliton microcomb generator. A tunable laser (NKT Koheras BASIK E15) is used as the pump laser. The pump is amplified by using

a high power / low noise EDFA (erbium doped fiber amplifier, Max-ray photonics, EYDFA-HP-C-BA-30-B) through a FPC (fiber polarization controller) and then launched into one side of the microring cavity through an FBG (Fiber Bragg grating) and a circulator in turn. Meanwhile, another tunable laser (PPCL 300) is used as the auxiliary laser for thermal stabilization, which is launched into the microcavity via the backward direction. We use a TEC (thermal electrical cooler, Thorlabs TC-200) to accurately control the macroscopic temperature, with a resolution of 10 mK. The generated combs are measured in OSA (optical spectrum analyzer, Yokogawa 6370D) and ESA (electrical spectrum analyzer, R&S 0 ~ 43 GHz). By carefully tuning the frequencies of the auxiliary and pump lasers, we obtain extremely stable solitons. The pump scanning can automatically operate via an upper controller. For suppressing the EDFA induced ASE noise, the filtering (via FBGs) is essential. **Fig. S5B** compares spectra of the soliton microcombs, generated in the system with and without using the FBG filters. It is clear that by using a narrow filter after the EDFA, the ASE noise induced by the amplification could be well suppressed. **Fig. S5C** presents the evolution of the intracavity power as a function of laser detuning, revealing a series of discrete steps associated with soliton formation. Here state I-V makes the diverse comb operations, ranging from chaotic state to single soliton. **Fig. S5D** shows the spectral evolution during the pumping laser scan. The formation of dissipative Kerr soliton involves Turing state (**I**), primary comb (**II**), modulation instability state, multi-soliton state (**III, IV**) and single-soliton state (**V**) gradually. The inset shows the wave-shape of soliton pulses in time-domain (about 340 fs) measured by using a frequency-resolved optical gating (Mesa Photonics FROG Scan), as **Fig. S5E** shows. After filtering & amplifying, a single pulse of the 10 comb lines (span 0.98 THz) is wider. Autocorrelation width is 3.6 ps, suggesting the single pulse width t_c = 2.33 ps.

In **Fig. S6**, we present additional results related to the locking of the pump laser and the dual comb repetitions. **Fig. S6A** shows the method schematically. By utilizing a broadband ultra-stable optical reference, we mix the optical beat notes with signals generated by RF references, subsequently establishing feedback loops onto the pump and auxiliary lasers (*50*). Once locked, the phase noise of each frequency channel is suppressed. Specifically, to lock the pump frequency (f_p), the repetition frequency of the probe comb (D_1), and the repetition frequency of the reference comb (D_1+ΔD_1), one requires three feedback loops. **Fig. S6B** depicts the single sideband frequency noise of the pump laser both before and after locking. For example, at a 1 Hz offset, the free-running laser exhibits a frequency noise of 2×10^8 Hz^2/Hz. After locking, the frequency noise at a 1 Hz offset reduces to 10^6 Hz^2/Hz. The black dashed line represents the noise base of the RF reference. The locking operation notably reduces the laser noise when the frequency offset is less than 10 kHz. In **Fig. S6C**, we display the PSD of our DAS (with a 1 kHz acoustic wave) using the single-frequency laser before and after locking. Thanks to the laser stabilization, noise levels are diminished. The improvement in SNR is more pronounced in the low-frequency region. In relation to noise suppression in the laser, the noise suppression ratio in the DAS is lower, primarily constrained by the system instability.

In **Fig. S7**, we show the device based limitations. **Fig. S7A** shows the spectra of two frequency combs schematically. There is a smaller frequency offset (Δf_p) between the probe comb's pump and the reference comb's pump. For the comb lines located at the right side of the pumps, beating frequencies are $\Delta f_p + N\Delta D_1$. For the comb lines at the left side of the pumps, beating frequencies are $-\Delta f_p + N\Delta D_1$. To avoid aliasing in the RF, it is a common practice to just use one side (left or right). In our experiment, we use the frequency comb lines on the right side. Specifically, Δf_p = 5 MHz, ΔD_1 = 200 MHz. Then, all the comb lines must be located within the amplification band of the EDFA, which is shown in **Fig. S7B**. The effective amplification band is 1525 nm ~ 1564 nm. When considering combs with pump frequency ≈ 1550 nm, the useful amplification bandwidth is < 17 THz. Considering D_1 = 110 GHz, it can accommodate up to 16 comb lines. If one wants to use more comb lines, one solution is to use a higher pump frequency (e.g. @ 1530 nm). But this may ask for redesigning the microcavity dispersion. Another way is to use a smaller D_1, but this means one needs to use microresonators with larger cavity length. Besides, the photodetector can

also limit the number of comb lines. **Fig. S7C** shows the response of our low noise balanced photodetector (PDB482C-AC). When more comb lines are needed, one can use a photodetector with broader bandwidth (but commonly the noise of the photodetector increases with its bandwidth), or decrease the ΔD_1 (that means the spatial resolution would be sacrificed).

Figure S8 shows more details about the single comb line based DAS measurement. In order to further better verify the effectiveness of comb DAS, we also use one single comb line (the first comb line away from the pumping frequency) in the 10 km sensing experiment, and compare the measured results with the single frequency laser DAS based on the NKT-E15 source. **Fig. S8A** plots the optical spectrum and the linewidth of the first soliton comb line at ≈ 1552 nm. The linewidth of the comb line is 160 Hz, similar to the pumping laser (NKT E15). Using this comb line as the source, we repeat the sensing in the same setup and scheme (10 km, resolution 1 m as the maintext shows). **Fig. S8B** shows the sensing results. For 10 Hz, 100 Hz and 1000 Hz acoustic signals, the single comb line demonstrates a similar response to a single frequency laser with the same power. **Fig. S8C** and **S8D** compares their sensitivities, verifying that every comb line has comparable performance to a single frequency laser. The spectral noise also suggests that using a single comb line or using a single frequency laser can achieve nearly equal resolution.

To further verify the dual-comb based sensing enhancement, we measure more cases. In **Fig. S8E to S8F**, we employed a 10 km (1 m spatial resolution) setup to sense the acoustic signals generated by a PZT at 5 km away (PZT voltage 1 V, frequency 100 Hz, sampling rate 10 GHz). **Fig. S8E** demonstrates the measured temporal $\Delta\varphi$, from left to right, we use one, two, five, eight and ten comb lines. It is clear that with increasing the comb line number, SNR of the sensing result improves. **Fig. S8F** plots the corresponding power spectra of the measured acoustic signals. The peak power in all cases is normalized at the 0 dBc. By using one, two, five, eight and ten comb lines, the SNR of PSD increases from 29 dB to 35 dB, 43 dB, 47 dB and 49 dB. Such results illustrate the linear boosting of sensitivity well.

Besides sensitivity enhancement, the utilization of the soliton comb source with multiple coherent frequencies can suppress the coherent fading. In conventional DAS using one single laser frequency, destructive interferences at random positions are inevitable. To solve this problem, a common strategy is to use multiple frequencies (*53*). Our soliton microcomb naturally outputs multiple frequencies without the need for additional modulation, offering unique simplicity. **Fig. S9A** compares the intensity of Rayleigh scattering signal in our DAS, when the launched-in comb number increases from 1 to 10. Statistically, normalized Rayleigh scattering signal (RBS) intensity is shown in **Fig. S9B**. An obvious result is that after using 10 comb line, the number of severe fading points (normalized intensity <0.6) decreases from 53 to 2.

As mentioned in the main text, the dual-comb scheme provides a way enabling higher energy, which helps for longer distance sensing. Here we show more details that how we use the dual comb source detecting acoustic signals 100 km away. System parameters are given in the main text, here we use another acoustic signal (PZT voltage 10 V, frequency 50 Hz). First, we confirm that by using a single frequency laser below the Brillouin threshold, we cannot see any response due to the fiber loss. **Fig. S9C** shows the schematic setup of this case, **Fig. S9D** plots the detected $\Delta\varphi$ trace, **Fig. S9E** shows the detected power spectrum of the acoustic signal. Just using a single frequency source (NKT E15), an acoustic signal cannot be detected at a distance of 100 km. On the other hand, we illustrate the result when using the 10-line dual comb source. **Fig. S9F** shows the scheme schematically. In **Fig. S9G**, we obtain the $\Delta\varphi$ trace of the dual-comb based DAS, despite the presence of slow energy fluctuation, the sinusoidal response is clear, with a fixed period 0.02s. Hence, we plot its power spectrum via FFT, in **Fig. S9H**. An obvious peak at 50 Hz suggests the successful acoustic sensing. It also demonstrates SNR > 10 dB. The obtained sensing results accurately capture the operation of the PZT located at 100 km away.

Besides the packaged chips and the automatically generated soliton microcombs (**Fig. S5**), the whole dual soliton microcomb source can be instrumentally packaged for potential out-of-lab

applications. **Fig. S10A** shows the picture of our plug-and-play dual soliton microcombs, which can be encapsulated in a 50 cm × 50 cm × 20 cm chassis. Here we have integrated the pump lasers, auxiliary lasers, temperature controllers, feedback devices, polarization controllers, and filtering/shaping devices together. In the setup, every single comb device is packaged in a metal box, which links a thermos-electrical cooler (TEC). The chip-fiber coupling is realized by using lens fiber ends. **Fig. S10B** plots the Allan deviation of the dual soliton beat signal. Frequency uncertainty reaches 6×10^{-10} when the average delay is 0.2 s. In laboratory environment, we measure the stability of the dual comb source in 3 hours, as shown in **Fig. S10C**, both the probe comb and the reference comb can keep the locking state. We also note that when suddenly experiencing environment fluctuations such as severe temperature change or vibration, the soliton state may collapse. In the feedback loop, we trigger the sharp power drop, then the program will rescan the pump frequency, and automatically restart the dual comb source (**Fig. S10D**).

Supplementary Note S3. Performance comparisons

In this section we compare the performance of our scheme with recent advances. First, we discuss the 'comb-like' multi-frequency-based DASs. Since 2020, researchers investigating fiber DAS technology have shown interests in comb-like laser sources. But before our work, most of the literature just used electro-opto comb sources, which are realized by using modulators. **Table S2** shows several examples. Up to now, the use of modulators has allowed for the generation of multiple frequencies for DAS, and the spatial resolution could be improved due to the time-expansion effect. But for remarkable sensitivity enhancement, phase-locked dual soliton microcombs have unique advantages.

Table S2. DAS using comb like sources

Ref.	Source	Detection limit	Sensing distance	Spatial resolution
(*54*)	Negative sidebands	97 pε	103 km<br>Using distributed Raman amplification	9.3 m
(*44*)	Electro-optic comb	350 nε	1 km	4 cm
(*55*)	Electro-optic comb	770 nε	2 km	2 cm
**This work**	Dual soliton microcombs	560 fε	72 km<br>Without distributed amplification	5 m

As mentioned in the main text, integrated dual soliton microcombs are naturally suitable for DAS, while the stability of a comb source is one of the most important factors. In **Table S3**, we compare the intensity noise and the phase noise of our dual soliton microcombs with other advances. For acoustic sensing, phase noise in the 0 ~ 1 kHz range is a major limitation. Stability of our dual soliton microcombs is comparable to the state-of-the-art devices.

Table S3. Dual soliton microcombs

Ref.	Beat SNR	Beat phase noise	Min Allan deviation
(*34*)	60 dB	-88 dBc/Hz @ 1 kHz	Not given

(*36*)	60 dB	-50 dBc/Hz @ 1 kHz	Not given
(*39*)	50 dB	-90 dBc/Hz @ 1 kHz	2×10^{-7} @ 0.01 s
(*47*)	66 dB	-82 dBc/Hz @ 1 kHz	Not given
(*56*)	Not given	-80 dBc/Hz @ 1kHz	Not given
(*57*)	60 dB	-50 dBc/Hz @ 1 kHz (estimated)	Not given
(*58*)	> 40 dB	-75 dBc/Hz @ 1 kHz	5×10^{-10} @ 1 s
**This work**	60 dB	-89.8 dBc/Hz @ 1 kHz	6×10^{-10} @ 0.1 s

Table S4 compares various high-sensitivity DAS schemes. In recent years, by using chirped pulse modulation, Rayleigh scattering enhanced fibers, and deep learning strategies, the sensitivity of fiber DAS increases rapidly (*2*)(*59*). Before this work, the world record of DAS sensitivity was 600 fε/√Hz, achieved by the US Naval Research Laboratory (*60*). In this literature, scientists suggested that coherent DAS based on frequency synthesis measurement is a promising research direction. Besides, we note that quasi distributed sensing schemes (e.g. using fiber Bragg grating array) (*61*) have shown < 100 fε/√Hz sensitivity, but they are not fully distributed.

Table S4. Ultrahigh sensitivity DAS systems

Ref.	Sensitivity	Method
(*62*)	18 pε/√Hz	Scattering enhanced fiber
(*63*)	3.4 pε/√Hz	Chirped-pulse
(*64*)	2.58 pε/√Hz	LS-SVM assistance
(*65*)	1 pε/√Hz	Sampling error elimination
(*60*)	600 fε/√Hz	Coherent frequency-shifted pulses
**This work**	560 fε/√Hz	Dual soliton microcomb based parallel sensing

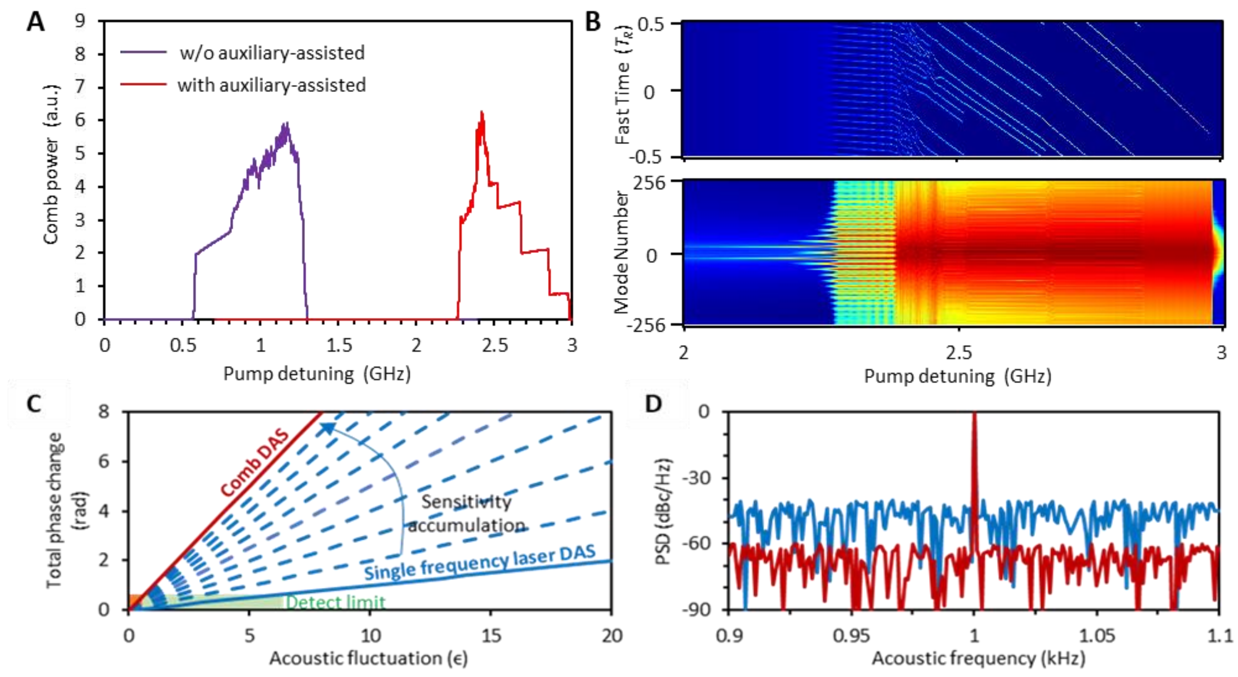

Fig. S1. Simulation of comb evolutions of auxiliary-assisted deterministic Kerr soliton generation and effect of sensitivity and SNR enhancement of the comb DAS. (A) Evolution of intracavity comb power trace with and without the auxiliary laser. The soliton states can be obtained more deterministically with the assistance of auxiliary laser. **(B)** Evolutions of the intracavity waveforms (left) and comb spectra (right) under auxiliary-assisted. **(C)** Conceptual calculation of the phase response accumulation due to the coherent frequency channel multiplexing. **(D)** Calculated result of the normalized PSD, blue: single frequency, red: 10-line comb with 10 folds intensity higher.

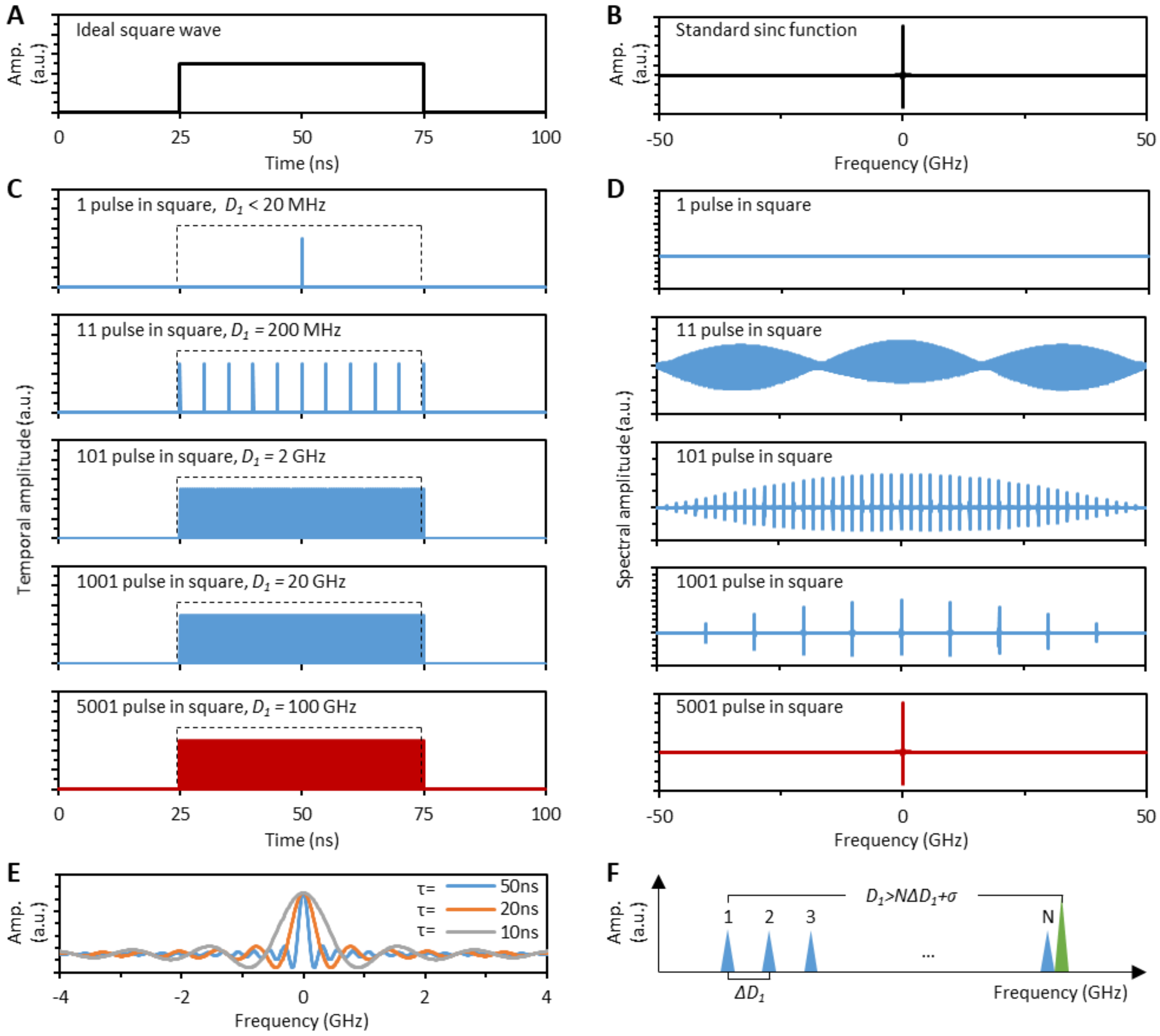

Fig. S2. Time-frequency relationship of square wave modulated comb pulses. (A) & (B) A typical 50 ns wide square pulse, its spectrum is in standard sinc shape. Experimental architecture of our dual-soliton generator. EDFA: erbium doped fiber amplifier. **(C) & (D)** When the pulse density increases in the square envelope, its spectrum evolves towards the idea sinc shape. **(E)** Bandwidth of the sinc spectrum is determined by the width of the square pulse. **(F)** For avoiding spectral aliasing, the comb repetition should be large enough.

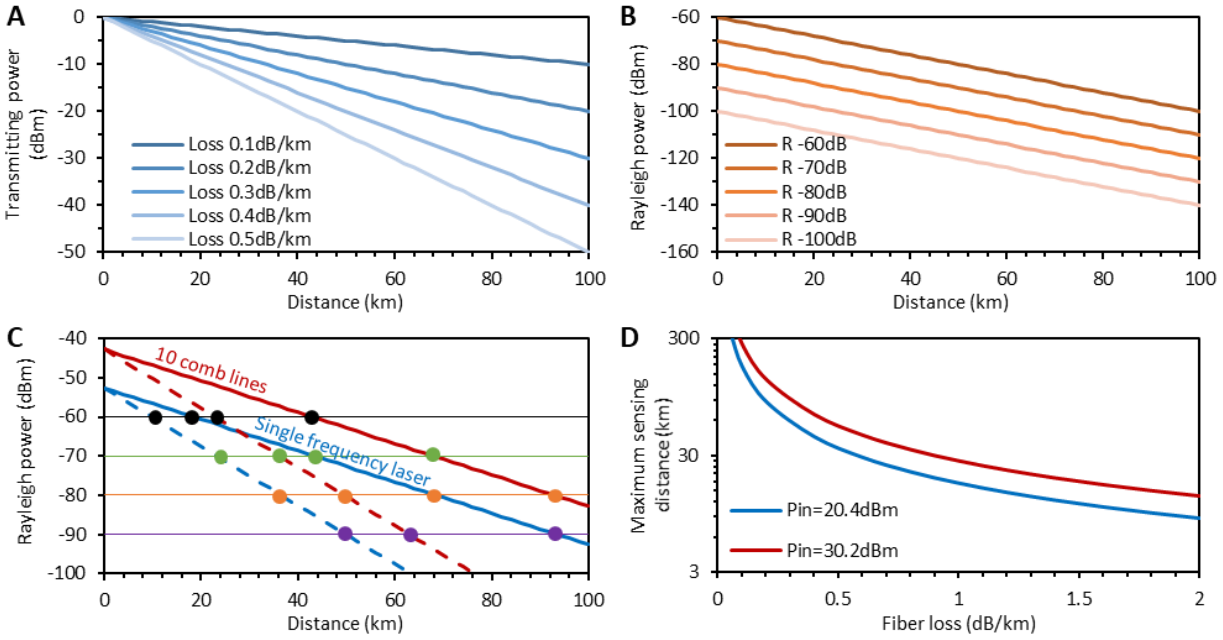

Fig. S3. Discussion of the sensing distance. (A) Calculated in-fiber power versus transmitting distance. **(B)** Calculated Rayleigh scattering power for different Rayleigh scattering coefficient, here transmitting loss is fixed (0.4 dB/km). **(C)** Maximum sensing distance locates at the intersection points of the power curves and photodetector detection limit lines. **(D)** Calculated maximum sensing distance, its inverse ratio is related to the fiber loss.

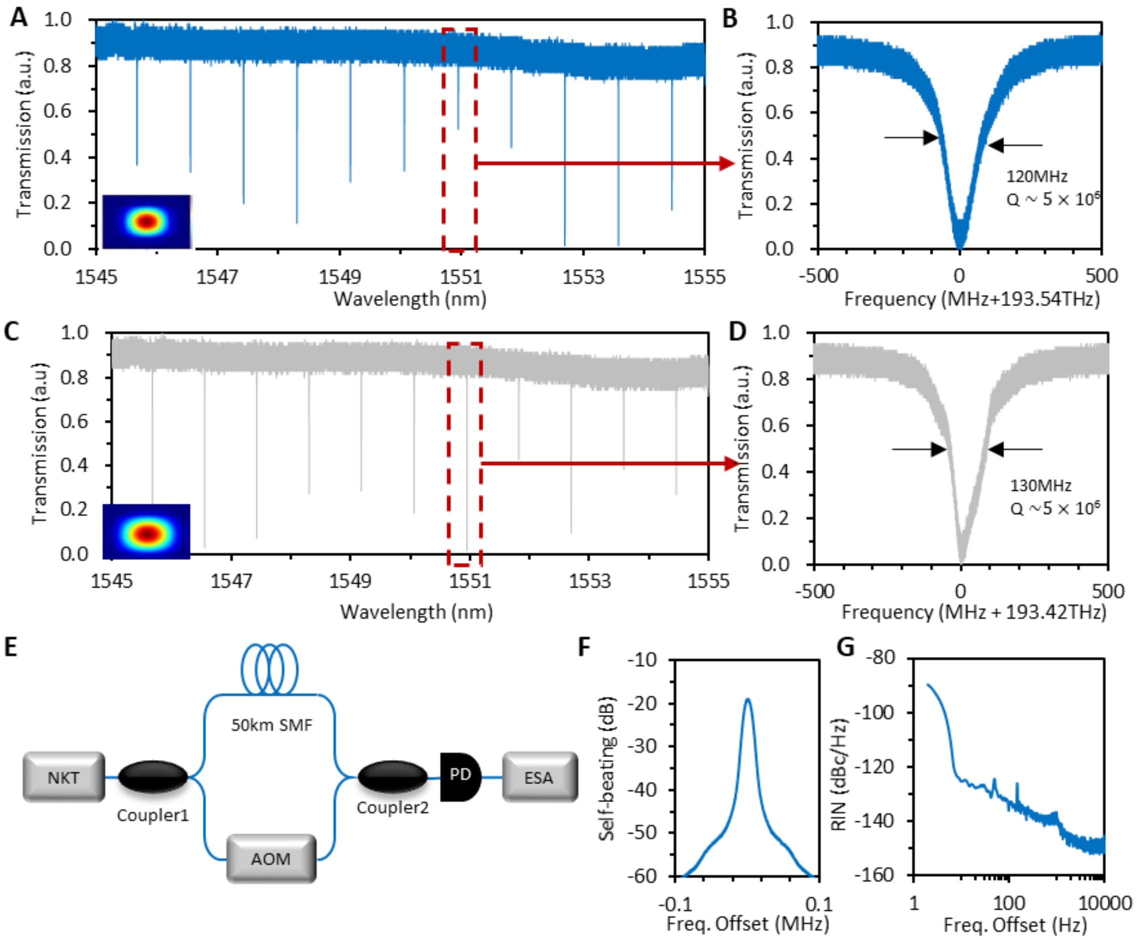

Fig. S4. Characterization of the microcavity and the NKT-E15. (A) The transmission spectrum of the Si_3N_4 microring cavity #1. **(B)** A single resonance (FWHM: 120MHz; Q factor ~ 10^6) of this cavity #1. **(C)** The transmission spectrum of the Si_3N_4 microring cavity #2. **(D)** A single resonance (FWHM: 130MHz; Q factor ~ 10^6) of this cavity #2. Insets: transverse electrical mode field distribution of the Si_3N_4 microring cavities. **(E)** Experimental setup for the optical linewidth measurement. FBG filter: Fiber Bragg Grating filter, AOM: acousto-optic modulator, PD: Photodetector, ESA: electrical spectrum analyzer. **(F)** Heterodyne self-beating note of the NKT E-15. **(G)** Relative intensity noise (RIN) of the NKT E-15.

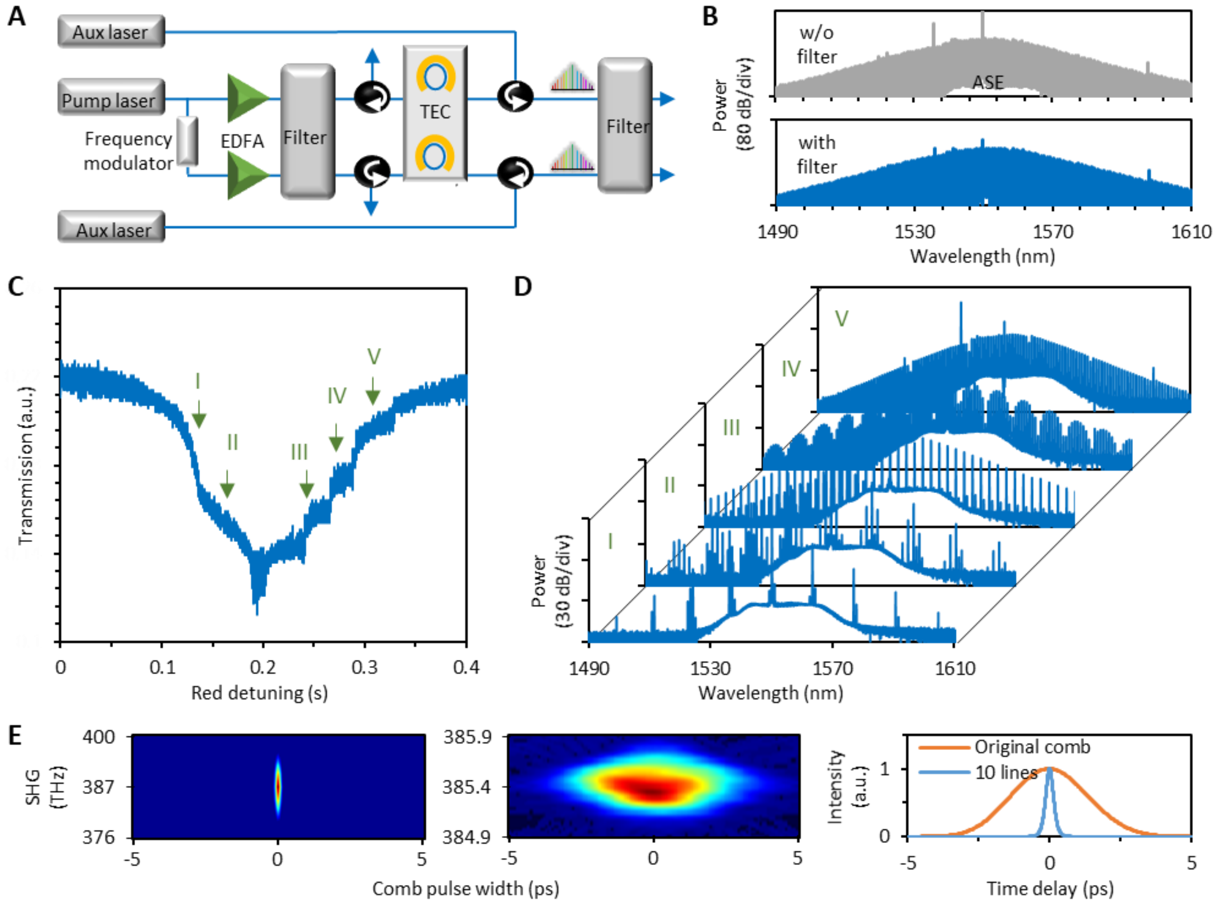

Fig. S5. DKS generation in the Si_3N_4 microring cavities. (A) Experimental architecture of our dual-soliton generator. **(B)** Spectra of a soliton microcomb, generated by an amplified pump laser without (grey) and with a filter. The filter contributes to suppress the ASE noise. **(C)** Measured transmitting optical intensity when scanning a laser over a resonance of the Si_3N_4 microring cavity (coupled pump power 50 mW). **(D)** Optical spectra of five different comb states during the pumping red-scan. Turing state (I), primary comb (II), multi-soliton state (III, IV) and single soliton state (V). Inset: an example FROG map of the DKS pulse. **(E)** Pulse characterization of the soliton comb.

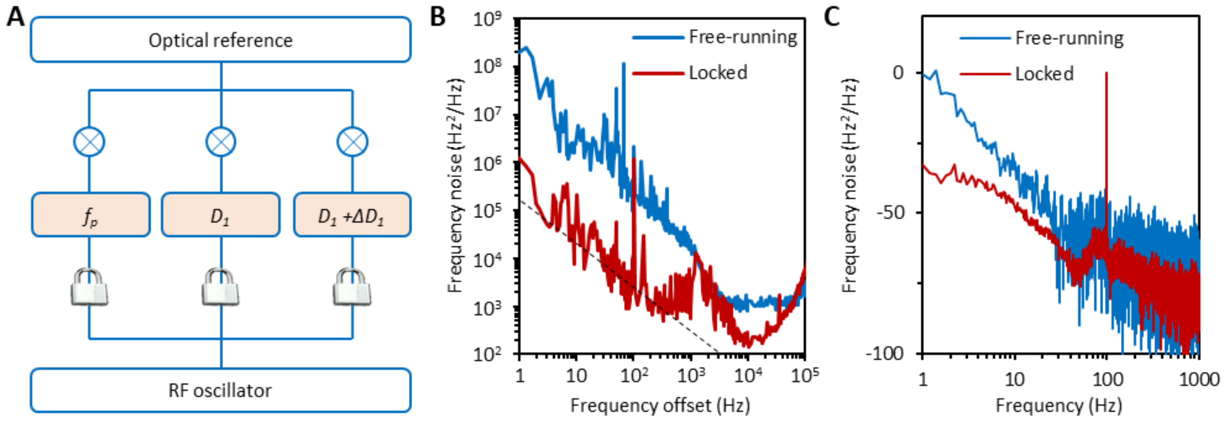

Fig. S6. Locking the laser. (A) Scheme of locking the laser frequency and the comb lines. **(B)** Frequency noise of the pump laser before and after locking. **(C)** Noise floor of the DAS based on the laser before and after locking.

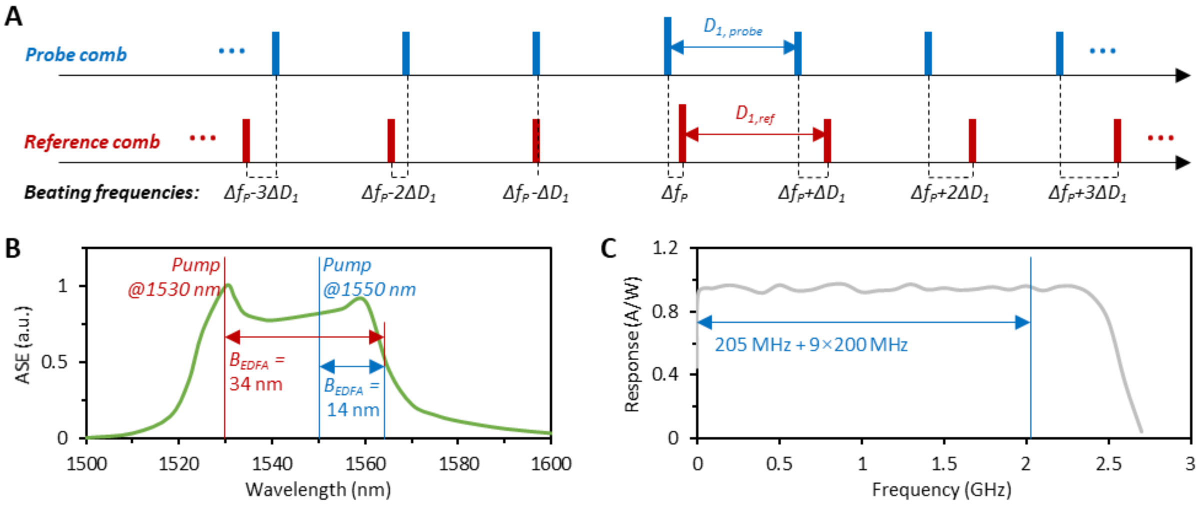

Fig. S7. Limitations from the devices. (A) Schematic spectra shows that the beating frequencies are different at the left and right sides of the pumps. **(B)** Amplification spectrum of our EDFA. **(C)** Bandwidth of our low noise photodetector.

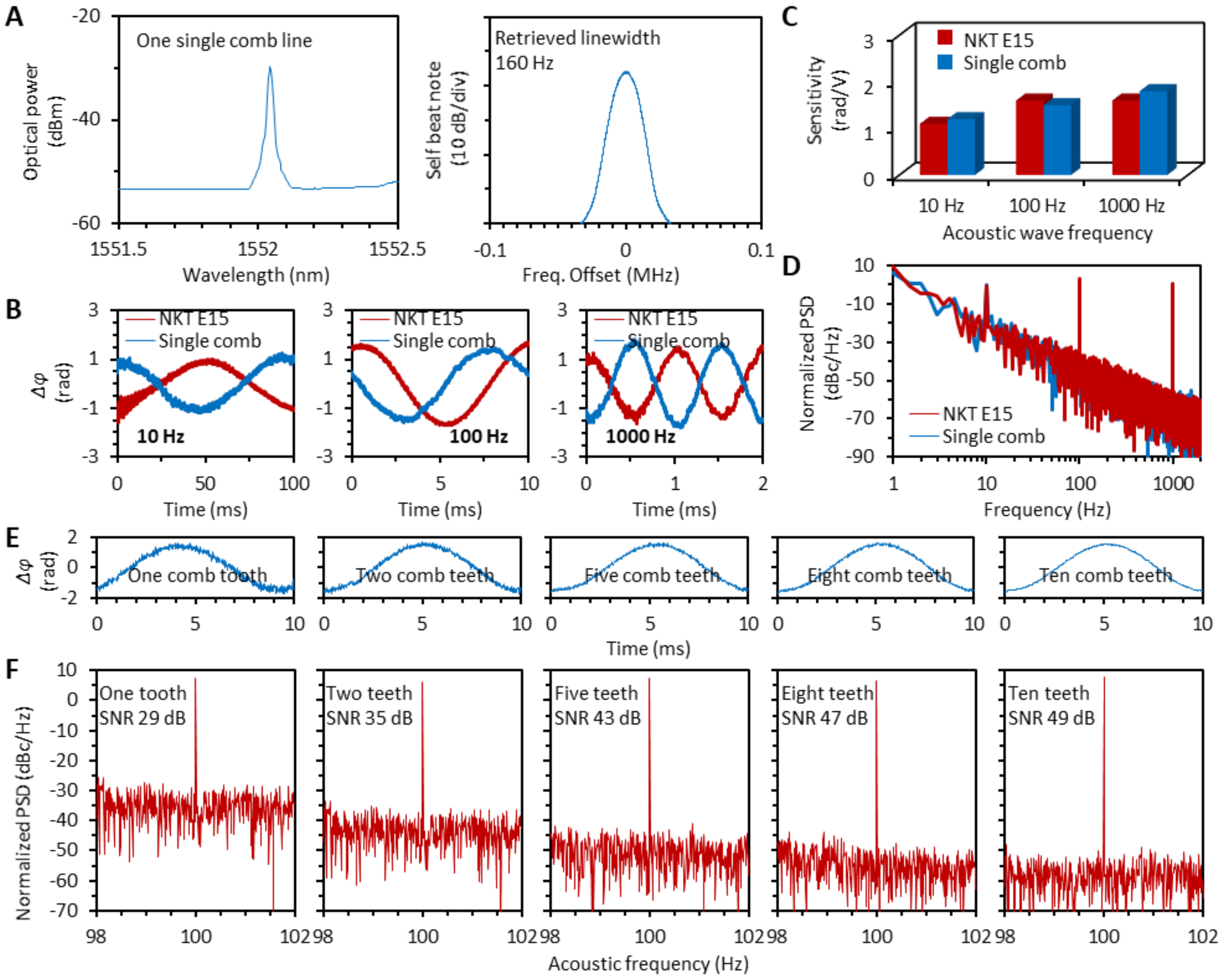

Fig. S8. Using one single soliton comb line and multiple soliton comb lines in DAS. (A) Optical spectrum and self-beat note of the single comb line. **(B)** In DAS, the measured $\Delta\varphi$ traces, when the acoustic frequency is 10 to 1000 Hz. **(C)** Sensitivity comparison of the NKT E15 based DAS and the single comb line based DAS. **(D)** PSD of the two sources based DAS, here the peaks are acoustic stimulations at varied frequencies. **(E)** Measured temporal traces of the acoustic signals. **(F)** Measured PSD of the acoustic signals. From left to right, we use one, two, five, eight and ten soliton comb lines for the DAS detection.

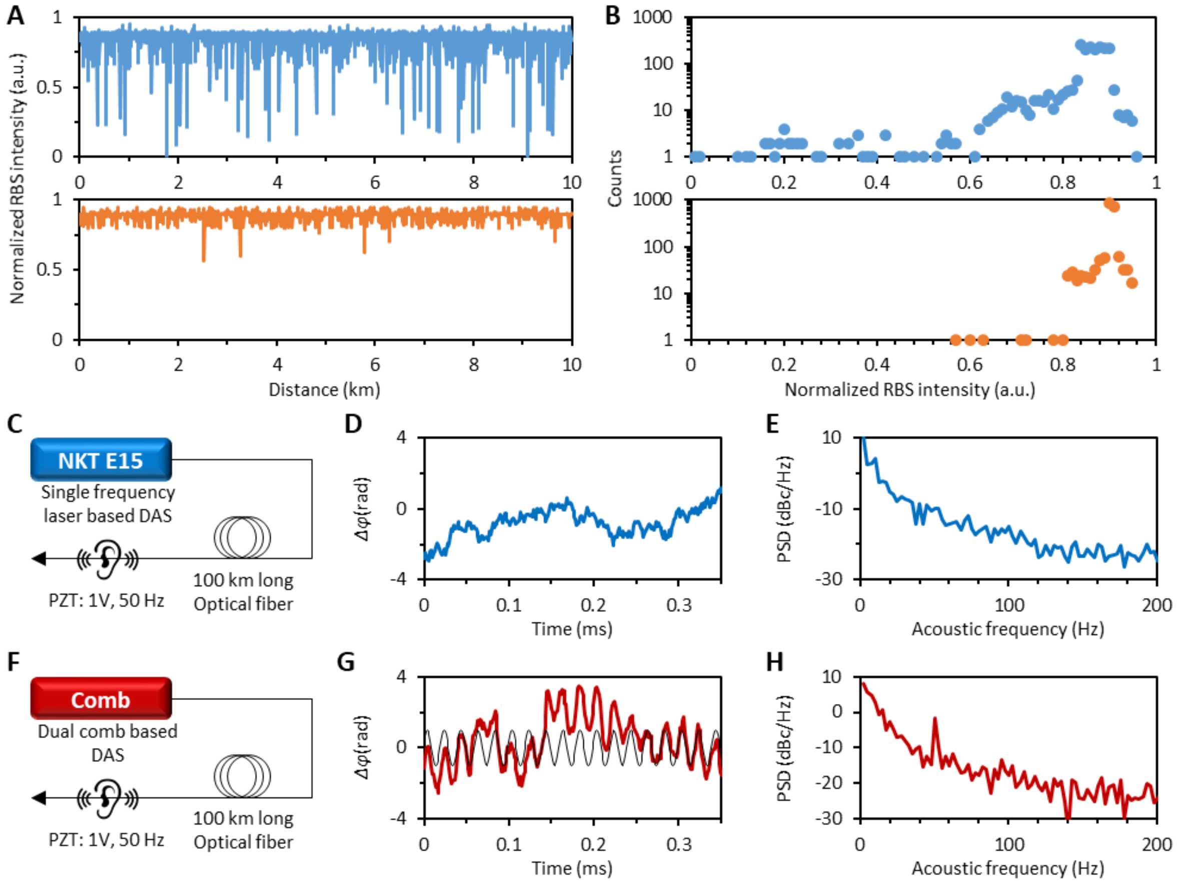

Fig. S9. Fading suppression and Extended results that we use the comb-DAS measuring acoustic response in a distance of 100 km. (A) Normalized RBS intensity when using a single frequency laser (top) and a 10-lines soliton comb (bottom). **(B)** Statistical counts of the RBS intensities, when using a single frequency laser (top) and a 10-lines soliton comb (bottom). **(C)** Schematic setup using a single frequency laser. **(D)** & **(E)** Temporal trace of $\Delta\varphi$ and power spectrum obtained by the single frequency laser based DAS. **(F)** Schematic setup using the dual-comb source. **(G)** & **(H)**, Temporal trace of $\Delta\varphi$ and power spectrum obtained by the dual-comb based DAS.

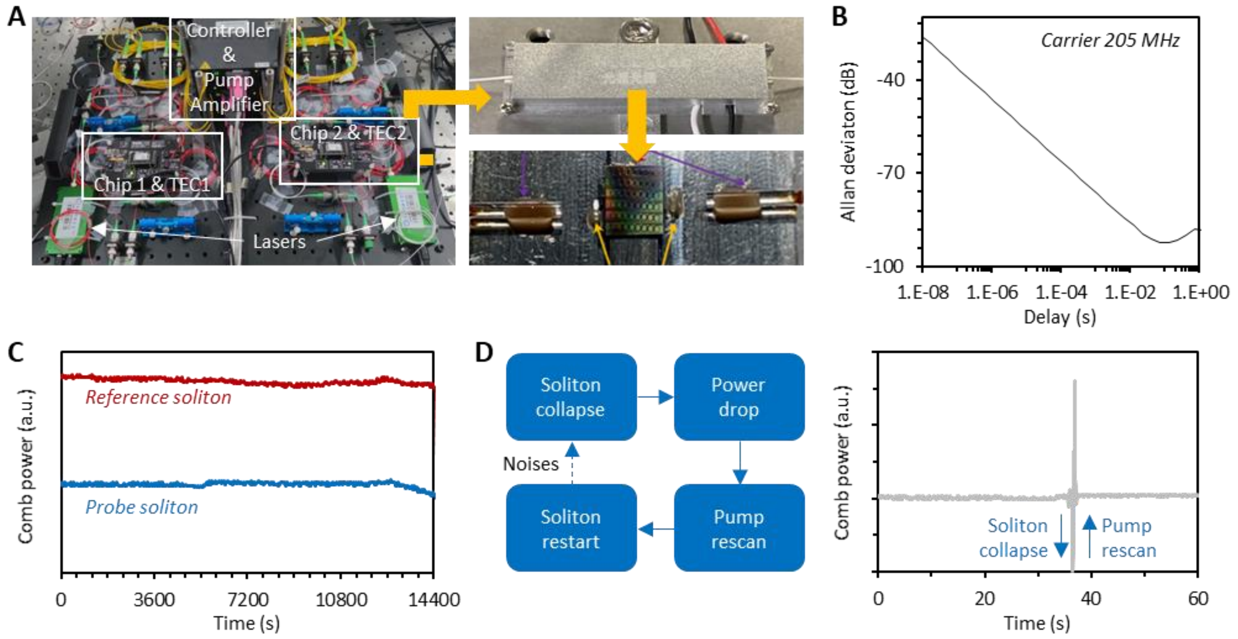

Fig. S10. Practicality of the dual microcomb instrument. (A) Picture of the whole dual comb system, the encapsulated microresonator device and its inner architecture. **(B)** Allan deviation of the dual soliton beat signal. **(C)** Power stability during 4 h. **(D)** When the comb collapses, the pump can rescan to recover the soliton automatically.

REFERENCES AND NOTES

1. A. H. Hartog, *An Introduction to Distributed Optical Fibre Sensors* (CRC, 2017).

2. Y. Rao, Z. Wang, H. Wu, Z. Ran, B. Han, Recent advances in phase-sensitive optical time domain reflectometry (Φ-OTDR). *Photonic Sens.* **11**, 1–30 (2021).

3. N. J. Lindsey, T. C. Dawe, J. B. Ajo-Franklin, Illuminating seafloor faults and ocean dynamics with dark fiber distributed acoustic sensing. *Science* **366**, 1103–1107 (2019).

4. A. Sladen, D. Rivet, J. P. Ampuero, L. De Barros, Y. Hello, G. Calbris, P. Lamare, Distributed sensing of earthquakes and ocean-solid Earth interactions on seafloor telecom cables. *Nat. Commun.* **10**, 5777 (2019).

5. J. Li, T. Kim, N. Lapusta, E. Biondi, Z. Zhan, The break of earthquake asperities imaged by distributed acoustic sensing. *Nature* **620**, 800–806 (2023).

6. F. Walter, D. Gräff, F. Lindner, P. Paitz, M. Köpfli, M. Chmiel, A. Fichtner, Distributed acoustic sensing of microseismic sources and wave propagation in glaciated terrain. *Nat. Commun.* **11**, 2436 (2020).

7. I. Ashry, Y. Mao, B. Wang, F. Hveding, A. Y. Bukhamsin, T. K. Ng, B. S. Ooi, A review of distributed fiber–optic sensing in the oil and gas industry. *J. Lightwave Technol.* **40**, 1407–1431 (2022).

8. B. Lu, B. Wu, J. Gu, J. Yang, K. Gao, Z. Wang, L. Ye, Q. Ye, R. Qu, X. Chen, H. Cai, Distributed optical fiber hydrophone based on Φ-OTDR and its field test. *Opt. Express***29**, 3147–3162 (2021).

9. J. M. Muggleton, R. Hunt, E. Rustighi, G. Lees, A. Pearce, Gas pipeline leak noise measurements using optical fibre distributed acoustic sensing. *J. Nat. Gas Sci. Eng.* **78**, 103293 (2020).

10. P. G. Hubbard, J. Xu, S. Zhang, M. Dejong, L. Luo, K. Soga, C. Papa, C. Zulberti, D. Malara, F. Fugazzotto, F. Martin-Lopez, C. Minto, Dynamic structural health monitoring of a

model wind turbine tower using distributed acoustic sensing (DAS). *J. Civ. Struct. Health Monit.* **11**, 833–849 (2021).

11. B. Han, H. Guan, J. Yao, Y. Rao, Z. Ran, Y. Gong, Q. Li, M. Li, R. Zhang, S. An, G. Yu, X. Wang, Distributed acoustic sensing with sensitivity-enhanced optical cable. *IEEE Sens. J.* **21**, 4644–4651 (2021).

12. F. Peng, H. Wu, X. Jia, Y. Rao, Z. Wang, Z. Peng, Ultra-long high-sensitivity Φ-OTDR for high spatial resolution intrusion detection of pipelines. *Opt. Express* **22**, 13804–13810 (2014).

13. X. Sun, Z. Yang, X. Hong, S. Zaslawski, S. Wang, M. A. Soto, X. Gao, J. Wu, L. Thévenaz, Genetic-optimised aperiodic code for distributed optical fibre sensors. *Nat. Commun.* **11**, 5774 (2020).

14. M. Galal, S. Sebastian, Z. Yang, L. Zhang, S. Zaslawski, L. Thévenaz, On the measurement accuracy of coherent Rayleigh-based distributed sensors. *Opt. Express* **29**, 42538–42552 (2021).

15. X. Lu, K. Krebber, Characterizing detection noise in phase-sensitive optical time domain reflectometry. *Opt. Express* **29**, 18791–18806 (2021).

16. T. J. Kippenberg, A. L. Gaeta, M. Lipson, M. L. Gorodetsky, Dissipative Kerr solitons in optical microresonators. *Science* **361** (2018).

17. L. Chang, S. Liu, J. E. Bowers, Integrated optical frequency comb technologies. *Nat. Photonics* **16**, 95–108 (2022).

18. B. Yao, S. Huang, Y. Liu, A. K. Vinod, C. Choi, M. Hoff, Y. Li, M. Yu, Z. Feng, D. Kwong, Y. Huang, Y. Rao, X. Duan, C. W. Wong, Gate-tunable frequency combs in graphene–nitride microresonators. *Nature* **558**, 410–414 (2018).

19. P. M. Palomo, J. N. Kemal, M. Karpov, A. Kordts, J. Pfeifle, M. H. P. Pfeiffer, P. Trocha, S. Wolf, V. Brasch, M. H. Anderson, R. Rosenberger, K. Vijayan, W. Freude, T. J. Kippenberg,

C. Koos, Microresonator-based solitons for massively parallel coherent optical communications. *Nature* **546**, 274–279 (2017).

20. B. Corcoran, M. Tan, X. Xu, A. Boes, J. Wu, T. G. Nguyen, S. T. Chu, B. E. Little, R. Morandotti, A. Mitchell, D. J. Moss, Ultra-dense optical data transmission over standard fibre with a single chip source. *Nat. Commun.* **11**, 2568 (2020).

21. Y. Geng, H. Zhou, X. Han, W. Cui, Q. Zhang, B. Liu, G. Deng, Q. Zhou, K. Qiu, Coherent optical communications using coherence-cloned Kerr soliton microcombs. *Nat. Commun.* **13**, 1070 (2022).

22. X. Xu, M. Tan, B. Corcoran, J. Wu, A. Boes, T. G. Nguyen, S. T. Chu, B. E. Little, D. G. Hicks, R. Morandotti, A. Mitchell, D. J. Moss, 11 TOPS photonic convolutional accelerator for optical neural networks. *Nature* **589**, 44–51 (2021).

23. J. Feldmann, N. Youngblood, M. Karpov, H. Gehring, X. Li, M. Stappers, M. L. Gallo, X. Fu, A. Lukashchuk, A. S. Raja, J. Liu, C. D. Wright, A. Sebastian, T. J. Kippenberg, W. H. P. Pernice, H. Bhaskaran, Parallel convolutional processing using an integrated photonic tensor core. *Nature* **589**, 52–58 (2021).

24. M. A. Guidry, D. M. Lukin, K. Y. Yang, R. Trivedi, J. Vučković, Quantum optics of soliton microcombs. *Nat. Photonics* **16**, 52–58 (2022).

25. M. Suh, X. Yi, Y. Lai, S. Leifer, I. S. Grudinin, G. Vasisht, E. C. Martin, M. P. Fitzgerald, G. Doppmann, J. Wang, D. Mawet, S. B. Papp, S. A. Diddams, C. Beichman, K. Vahala, Searching for exoplanets using a microresonator astrocomb. *Nat. Photonics* **13**, 25–30 (2019).

26. E. Obrzud, M. Rainer, A. Harutyunyan, M. H. Anderson, J. Liu, M. Geiselmann, B. Chazelas, S. Kundermann, S. Lecomte, M. Cecconi, A. Ghedina, E. Molinari, F. Pepe, F. Wildi, F. Bouchy, T. J. Kippenberg, T. Herr, A microphotonic astrocomb. *Nat. Photonics* **13**, 31–35 (2019).

27. J. Zhang, B. Peng, S. Kim, F. Monifi, X. Jiang, Y. Li, P. Yu, L. Liu, Y. Liu, A. Alù, L. Yang, Optomechanical dissipative solitons. *Nature* **600**, 75–80 (2021).

28. P. J. Marchand, J. Riemensberger, J. C. Skehan, J. Ho, M. H. P. Pfeiffer, J. Liu, C. Hauger, T. Lasser, T. J. Kippenberg, Soliton microcomb based spectral domain optical coherence tomography. *Nat. Commun.* **12**, 427 (2021).

29. X. Ji, X. Yao, A. Klenner, Y. Gan, A. L. Gaeta, C. P. Hendon, M. Lipson, Chip-based frequency comb sources for optical coherence tomography. *Opt. Express* **27**, 19896–19905 (2019).

30. D. T. Spencer, T. Drake, T. C. Briles, J. Stone, L. C. Sinclair, C. Fredrick, Q. Li, D. Westly, B. R. Ilic, A. Bluestone, N. Volet, T. Komljenovic, L. Chang, S. H. Lee, D. Y. Oh, M. G. Suh, K. Y. Yang, M. H. P. Pfeiffer, T. J. Kippenberg, E. Norberg, L. Theogarajan, K. Vahala, N. R. Newbury, K. Srinivasan, J. E. Bowers, S. A. Diddams, S. B. Papp, An optical-frequency synthesizer using integrated photonics. *Nature* **557**, 81–85 (2018).

31. S. B. Papp, K. Beha, P. D. Haye, F. Quinlan, H. Lee, K. J. Vahala, S. A. Diddams, Microresonator frequency comb optical clock. *Optica* **1**, 10–14 (2014).

32. Z. L. Newman, V. Maurice, T. Drake, J. R. Stone, T. C. Briles, D. T. Spencer, C. Fredrick, Q. Li, D. Westly, B. R. Ilic, B. Shen, M. G. Suh, K. Y. Yang, C. Johnson, D. M. S. Johnson, L. Hollberg, K. J. Vahala, K. Srinivasan, S. A. Diddams, J. Kitching, S. B. Papp, M. T. Hummon, Architecture for the photonic integration of an optical atomic clock. *Optica* **6**, 680–685 (2019).

33. J. Riemensberger, A. Lukashchuk, M. Karpov, W. Weng, E. Lucas, J. Liu, T. J. Kippenberg, Massively parallel coherent laser ranging using a soliton microcomb. *Nature* **581**, 164–170 (2020).

34. T. Tan, Z. Yuan, H. Zhang, G. Yan, S. Zhou, N. An, B. Peng, G. Soavi, Y. Rao, B. Yao, Multispecies and individual gas molecule detection using Stokes solitons in a graphene over-modal microresonator. *Nat. Commun.* **12**, 6716 (2021).

35. N. An, T. Tan, Z. Peng, C. Qin, Z. Yuan, L. Bi, C. Liao, Y. Wang, Y. Rao, G. Soavi, B. Yao, Electrically tunable four-wave-mixing in graphene heterogeneous fiber for individual gas molecule detection. *Nano Lett.* **20**, 6473–6480 (2020).

36. H. Zhang, T. Tan, H. Chen, Y. Yu, W. Wang, B. Chang, Y. Liang, Y. Guo, H. Zhou, H. Xia, Q. Gong, C. W. Wong, Y. Rao, Y. Xiao, B. Yao, Soliton microcombs multiplexing using intracavity-stimulated Brillouin lasers. *Phys. Rev. Lett.* **130**, 153802 (2023).

37. I. Coddington, N. Newbury, W. Swann, Dual-comb spectroscopy. *Optica* **3**, 414–426 (2016).

38. M. G. Suh, K. J. Vahala, Soliton microcomb range measurement. *Science* **359**, 884–887 (2018).

39. C. Qin, J. Du, T. Tan, B. Chang, K. Jia, Y. Liang, W. Wang, Y. Guo, H. Xia, S. Zhu, Y. Rao, Z. Xie, B. Yao, Co-generation of orthogonal soliton pair in a monolithic fiber resonator with mechanical tunability. *Laser Photonics Rev.* **17**, 2200662 (2023).

40. C. Bao, Z. Yuan, L. Wu, M. Suh, H. Wang, Q. Lin, K. J. Vahala, Architecture for microcomb-based GHz-mid-infrared dual-comb spectroscopy. *Nat. Commun.* **12**, 6573 (2021).

41. Y. Tong, X. Guo, M. Li, H. Tang, N. Sharmin, Y. Xu, W. N. Lee, K. K. Tsia, K. K. Y. Wong, Ultrafast optical phase-sensitive ultrasonic detection via dual-comb multiheterodyne interferometry. *Adv. Photonics Nexus* **2**, 016002 (2023).

42. J. T. Friedlein, E. Baumann, K. A. Briggman, G. M. Colacion, F. R. Giorgetta, A. M. Goldfain, D. I. Herman, E. V. Hoenig, J. Hwang, N. R. Newbury, E. F. Perez, C. S. Yung, I. Coddington, K. C. Cossel, Dual-comb photoacoustic spectroscopy. *Nat. Commun.* **11**, 3152 (2020).

43. C. Jin, N. Guo, Y. Feng, L. Wang, H. Liang, J. Li, Z. Li, C. Yu, C. Lu, Scanning-free BOTDA based on ultra-fine digital optical frequency comb. *Opt. Express* **23**, 5277–5284 (2015).

44. M. S. Amat, H. F. Martins, V. Durán, L. Costa, S. Martin-Lopez, M. Gonzalez-Herraez, M. R. Fernández-Ruiz, Time-expanded phase-sensitive optical time-domain reflectometry. *Light Sci. Appl.* **10**, 51 (2021).

45. Z. Wang, L. Zhang, S. Wang, N. Xue, F. Peng, M. Fan, W. Sun, X. Qian, J. Rao, Y. Rao, Coherent Φ-OTDR based on I/Q demodulation and homodyne detection. *Opt. Express* **24**, 853–858 (2016).

46. Z. Wang, B. Zhang, J. Xiong, Y. Fu, S. Lin, J. Jiang, Y. Chen, Y. Wu, Q. Meng, Y. Rao, Distributed acoustic sensing based on pulse-coding phase-sensitive OTDR. *IEEE Internet Things J.* **6**, 6117–6124 (2019).

47. Y. Geng, X. Han, X. Zhang, Y. Xiao, S. Qian, Q. Bai, Y. Fan, G. Deng, Q. Zhou, K. Qiu, J. Xu, H. Zhou, Phase noise of Kerr soliton dual microcombs. *Opt. Lett.* **47**, 4838–4841 (2022).

48. T. Herr, V. Brasch, J. D. Jost, C. Y. Wang, N. M. Kondratiev, M. L. Gorodetsky, T. J. Kippenberg, Temporal solitons in optical microresonators. *Nat. Photonics* **8**, 145–152 (2014).

49. H. Zhou, Y. Geng, W. Cui, S. Huang, Q. Zhou, K. Qiu, C. W. Wong, Soliton bursts and deterministic dissipative Kerr soliton generation in auxiliary-assisted microcavities. *Light Sci. Appl.* **8**, 50 (2019).

50. S. W. Huang, J. Yang, M. Yu, B. H. Mcguyer, D. L. Kwong, T. Zelevinsky, C. W. Wong, A broadband chip-scale optical frequency synthesizer at 2.7×10^{-16} relative uncertainty. *Sci. Adv.* **2**, e1501489 (2016).

51. M. Wu, X. Fan, Q. Liu, Z. He, Quasi-distributed fiber-optic acoustic sensing system based on pulse compression technique and phase-noise compensation. *Opt. Lett.* **44**, 5969–5972 (2019).

52. H. Gabai, A. Eyal, On the sensitivity of distributed acoustic sensing. *Opt. Lett.* **41**, 5648–5651 (2016).

53. J. Zhou, Z. Pan, Q. Ye, H. Cai, R. Qu, Z. Fang, Characteristics and explanations of interference fading of a φ-OTDR with a multi-frequency source. *J. Lightwave Technol.* **31**, 2947–2954 (2013).

54. J. Xiong, Z. Wang, Y. Wu, H. Wu, Y. Rao, Long-distance distributed acoustic sensing utilizing negative frequency band. *Opt. Express* **28**, 35844–35856 (2020).

55. M. S. Amat, H. F. Martins, V. Durán, P. Fermoso, S. Martin-Lopez, M. Gonzalez-Herraez, M. R. Fernández-Ruiz, Frequency stability requirements in quasi-integer-ratio time-expanded phase-sensitive OTDR. *J. Lightwave Technol.* **41**, 777–783 (2023).

56. N. J. Lambert, L. S. Trainor, H. G. L. Schwefel, Microresonator-based electro-optic dual frequency comb. *Commun. Phys.* **6**, 89 (2023).

57. Q. F. Yang, X. Yi, K. Y. Yang, K. Vahala, Counter-propagating solitons in microresonators. *Nat. Photonics* **11**, 560–564 (2017).

58. B. Wang, Z. Yang, X. Zhang, X. Yi, Vernier frequency division with dual-microresonator solitons. *Nat. Commun.* **11**, 3975 (2020).

59. Y. Shang, M. Sun, C. Wang, J. Yang, Y. Du, J. Yi, W. Zhao, Y. Wang, Y. Zhao, J. Ni., Research progress in distributed acoustic sensing techniques. *Sensors* **22**, 6060 (2022).

60. H. M. Ogden, M. J. Murray, J. B. Murray, C. Kirkendall, B. Redding, Frequency multiplexed coherent φ-OTDR. *Sci. Rep.* **11**, 17921 (2021).

61. H. M. Ogden, M. Beresna, T. Lee, B. Redding, Enhanced bandwidth distributed acoustic sensing using a frequency multiplexed pulse train and micro-machined point reflector fiber. *Opt. Lett.* **47**, 529–532 (2022).

62. J. Yao, B. Han, X. Jiang, S. Cao, Y. Fu, Y. Rao, Z. Ran, W. Wang, H. Guan, J. Long, Cladding softened fiber for sensitivity enhancement of distributed acoustic sensing. *Opt. Express* **29**, 8216–8222 (2021).

63. M. R. Fernández-Ruiz, L. Costa, H. F. Martins, Distributed acoustic sensing using chirped-pulse phase-sensitive OTDR technology. *Sensors* **19**, 4368 (2019).

64. T. Liu, H. Li, T. He, C. Fan, Z. Yan, D. Liu, Q. Sun, Ultra-high resolution strain sensor network assisted with an LS-SVM based hysteresis model. *Opto-Electron. Adv.* **4**, 200037 (2021).

65. L. Costa, H. F. Martins, S. Martin-Lopez, M. R. Fernandez-Ruiz, M. Gonzalez-Herraez, Fully distributed optical fiber strain sensor with 10−12ϵ/√Hz sensitivity. *J. Light. Technol.* **37**, 4487–4495 (2019).
